# Inhibitors of glucosamine-6-phosphate synthase as potential antimicrobials or antidiabetics – synthesis and properties

**DOI:** 10.1080/14756366.2022.2096018

**Published:** 2022-07-08

**Authors:** Joanna Stefaniak, Michał G. Nowak, Marek Wojciechowski, Sławomir Milewski, Andrzej S. Skwarecki

**Affiliations:** a Department of Organic Chemistry and BioTechMed Center, Gdańsk University of Technology, Gdańsk, Poland; b Department of Pharmaceutical Technology and Biochemistry and BioTechMed Center, Gdańsk University of Technology, Gdańsk, Poland

**Keywords:** Glucosamine-6-phosphate synthase, antibacterial agents, antifungal agents, diabetes, drug synthesis

## Abstract

Glucosamine-6-phosphate synthase (GlcN-6-P synthase) is known as a promising target for antimicrobial agents and antidiabetics. Several compounds of natural or synthetic origin have been identified as inhibitors of this enzyme. This set comprises highly selective l-glutamine, amino sugar phosphate or transition state intermediate *cis*-enolamine analogues. Relatively low antimicrobial activity of these inhibitors, poorly penetrating microbial cell membranes, has been improved using the pro-drug approach. On the other hand, a number of heterocyclic and polycyclic compounds demonstrating antimicrobial activity have been presented as putative inhibitors of the enzyme, based on the results of molecular docking to GlcN-6-P synthase matrix. The most active compounds of this group could be considered promising leads for development of novel antimicrobial drugs or antidiabetics, provided their selective toxicity is confirmed.

## 1. Introduction – the target

l-Glutamine:d-fructose-6-phosphate amidotransferase, also known under the name of glucosamine-6-phosphate synthase (GlcN-6-P synthase), is a ubiquitous enzyme of primary anabolism, present in almost all known living organisms and tissues and known under the abbreviated name of GFA or GFAT. The reaction catalysed by GlcN-6-P synthase constitutes the first committed step of a branch of glycolysis, leading to the ultimate formation of 5′-diphospho-*N*-acetyl-D-glucosamine (UDP-GlcNAc), known as the hexosamine biosynthesis pathway (HBP). In mammals, the HBP has been identified as one of the biochemical pathways that could contribute to insulin resistance, which is a molecular basis of type-2 diabetes^1^. The elevated activity of GlcN-6-P synthase was found to be correlated with insulin resistance, postprandial hyperglycaemia and diabetic complications. In consequence, the human enzyme is considered a potential diabetes target^2–4^.

The human GlcN-6-P synthase, i.e. hGFAT, has been also assigned a prominent role in the close relationship between HBP and cancer. In this respect, it is worth mentioning that in humans, two GFAT paralogs exist, namely hGFAT1 encoded by the *gfpt1* gene and *gfpt2*-encoded hGFAT2, that primarily differ in their tissue-specific expression patterns^5^. Expression of *gfpt1* was found to be upregulated in breast^6^, prostate^7^ and hepatic^8^ cancers, while *gfpt2* is considerably overexpressed in pancreatic adenocarcinoma^9^, colorectal cancer^10^ and non-small-cell lung cancer^11^. Inhibition of GFPT2 selectively reduced KRAS/LKB1 co-mutant tumour cell growth in culture, xenografts and genetically modified mice^12^. The GlcN-6-P synthase inhibitor: nanoparticle conjugates were found to exhibit remarkable cytotoxicity against human cervical cancer (HeLa) and hypopharyngeal carcinoma cell lines^13^. Moreover, an inhibitor of GlcN-6-P synthase in combination with the established anticancer agent, cisplatin, demonstrated a synergistic effect^14^. Therefore, GlcN-6-P synthase is also considered a possible target for anticancer agents, at least in some cancer types.

On the other hand, in prokaryotic and eukaryotic microorganisms, cell walls of which are composed of amino sugar-containing macromolecules, like peptidoglycan and lipopolysaccharides in bacteria or chitin and *N*-glycosylated mannoproteins in fungi, GlcN-6-P synthase is an enzyme of crucial importance for cell survival and growth. Deletion of the GlcN-6-P synthase encoding gene in fungi and bacteria is lethal^15^^,^^16^ and even a short-term inhibition of GlcN-6-P synthase activity in cell wall containing microorganisms results in fungicidal or bactericidal effect. On the other hand, short-term inhibition of GlcN-6-P synthase activity in mammals is not lethal^17^ and in the case of infectious diseases in diabetic patients could be even beneficial, so the potential of this enzyme as a target for antibacterials and antifungals is unquestionable^18^.

**Scheme 1.**
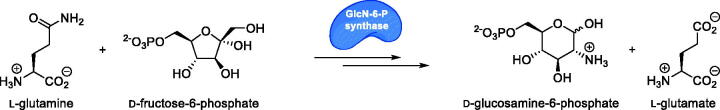
The reaction catalysed by GlcN-6-P synthase.

GlcN-6-P synthase catalyses an irreversible reaction between l-glutamine and d-fructose-6-phosphate, resulting in the formation of D-glucosamine-6-phosphate and L-glutamate (Scheme 1). The enzyme is a member of the so-called amidotransferase subfamily of enzymes, transferring amide nitrogen from L-glutamine to an acceptor substrate but is highly specific for its substrates, especially L-Gln. This specificity makes GlcN-6-P synthase unique among other enzymes of the subfamily, which are able to use ammonia as an alternative amino donor^19^^,^^20^.

**Figure 1.**
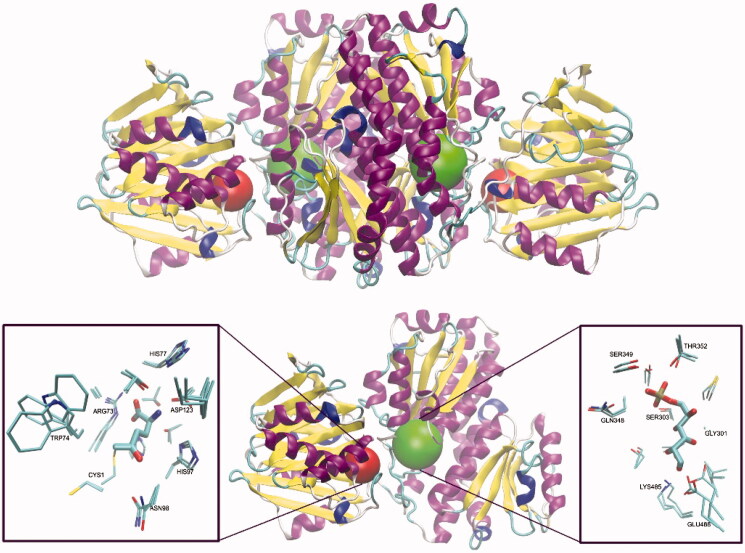
Molecular structure of GlcN-6-P synthase. Top – Structure of *E. coli* GFA dimer, with GAH and ISOM active centres indicated as red and green spheres, respectively. Based on the pdbid: 1jxa matrix. Bottom – a single subunit of GlcN-6-P synthase, with a detailed presentation of active centres’ crucial residues and 5-oxo-L-norleucine covalently bound to the Cys1 residue at GAH and Glc-6-P in an open ring form at ISOM. Side chains of residues present within the radius of 4.5 Å of both ligands are drawn as thin sticks and ligands as thicker sticks. Crucial residues of superimposed *C. albicans* (pdbid: 2poc) and *H. sapiens* (pdbid: 6r4f) GlcN-6-P synthases are shown, to visualise the cross-species conservation of both the structure and conformation of the crucial amino acid residues. The observed significant variations of Cys1, Trp74 and Glu488 confirmations are due to their conformational mobility during the catalytic act.

GlcN-6-P synthase is a relatively large, dimeric or tetrameric protein. Its monomeric subunit containing 589–716 amino acid residues^18^, is composed of two domains, the N-terminal domain (GAH) involved in L-Gln binding and hydrolysis and the d-Fru-6-P -binding C-terminal isomerase domain (ISOM). The prokaryotic GlcN-6-P synthase is a dimer of two identical subunits, as shown in Figure 1, while the eukaryotic enzyme is homotetrameric^18^. The prokaryotic (bacteria) and eukaryotic (fungi, mammals) enzyme versions differ also in terms of physiological modes of regulation of catalytic activity. In bacteria, expression of the GlcN-6-P synthase encoding gene is regulated posttranscriptionally by *si*RNA^21^, whereas the eukaryotic enzyme is a subject of allosteric feedback inhibition by UDP-GlcNAc^22^^,^^23^ and protein kinase A-mediated phosphorylation^24^.

**Scheme 2.**
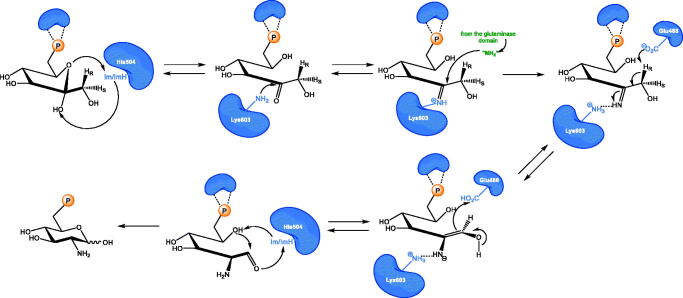
Mechanism of sugar phosphate isomerisation by GlcN-6-P synthase. Im and ImH^+^ represent the non-protonated or protonated form of the imidazole ring, respectively.

In GlcN-6-P synthase, there is not any single defined active centre but two active centres located at GAH and ISOM domains, respectively, are connected through the intramolecular, solvent inaccessible channel^25^. The only catalytic residue at GAH, namely N-terminal Cys1, catalyses the hydrolysis of l-Gln amide and three residues, namely Glu488, His504 and Lys603 (*E. coli* GlcN-6-P synthase numbering), participate in ketose-aldose isomerisation of fructosamine-6-P intermediate at ISOM^26^. All the catalytic residues and another five involved in substrate binding are highly conserved among GlcN-6-P synthases of different sources^18^. The molecular mechanism of GlcN-6-P synthase catalytic action is complex and involves three main steps: hydrolysis of glutamine at GAH, transfer of ammonia from GAH to ISOM and isomerisation of the resulting fructosamine-6-P at ISOM. At first, the Fru-6-P molecule binds to ISOM and the opening of its hexose ring triggers the conformational changes of two domains, namely closing access to the ISOM active site and promoting rearrangement of Cys1 at GAH into an active conformation. The subsequent binding of l-Gln at GAH induces another conformational change of the enzyme molecule, which ensures hydrolysis of glutamine amide and ammonia transfer through the intramolecular channel to ISOM. In the third step, the fructosamine-6-P is isomerised through the *cis*-enamine intermediate and finally, the reaction products, i.e. GlcN-6-P and l-Glu are released (Scheme 2)^27^.

## 2. Search for the GlcN-6-P synthase inhibitors

Due to the target potential of GlcN-6-P synthase, an extensive search for its inhibitors as potential antimicrobials or antidiabetics has been continued for several years. A number of such compounds have been found in Nature or synthesised as rationally designed molecules. Some of them or their derivatives exhibited expected biological activity. In another approach, several heterocyclic compounds exhibiting antimicrobial activity have been found potential GlcN-6-P synthase inhibitors by molecular docking studies. GlcN-6-P synthase inhibitors known so far fall into four groups: substrate analogues, transition state or intermediate analogues, product analogues and compounds binding outside the active centre. Some of them are alkylating agents, mechanism-based suicide inhibitors or transition metal complexes. Herein, we would like to present a comprehensive and concise review of the most significant examples of the GlcN-6-P synthase inhibitors, particularly focussing on their syntheses and antimicrobial or antidiabetic properties.

## 3. Glutamine analogues targeting GlcN-6-P synthase

**Scheme 3.**
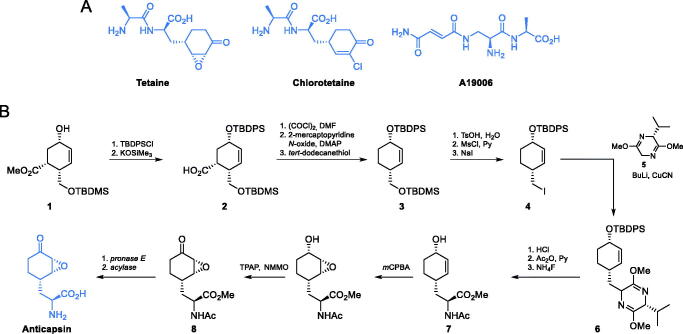
(A) Antibiotics containing glutamine analogues targeting GlcN-6-P synthase. (B) Synthesis of anticapsin reported by Baldwin *et al*. ^36^

Tetaine, also known under the name of bacilysin (Scheme 3A), is a natural compound produced by *Bacillus subtilis*^28^ that exhibits both antibacterial and antifungal activity^29^. The C-terminal amino acid of this dipeptide, anticapsin (Scheme 3B), produced independently by *Streptomyces griseoplanus*^30^, was identified as an irreversible inhibitor of GlcN-6-P synthase (*K*_i_ = 9.5 µM)^31^. This antimetabolite acts as a structural analog of l-glutamine and binds to the enzyme active site *via* alkylation of the catalytic Cys1 residue by its epoxide moiety^31^. In a tetaine analogue, known under the name of chororotetaine (Scheme 3A), the anticapsin residue is replaced by a structurally related, another GlcN-6-P synthase targeting glutamine analogue, containing a chlorocyclohexenone ring^32^.

In the structure of anticapsin, an *E* configuration was mistakenly assigned to the epoxide ring^33^ and corrected for the proper *Z* configuration two decades later^34^. Due to that error, there are reports before the year 1993 which claim to be synthetic methods of anticapsin preparation, but in reality concerned synthesis of its enantiomer^35^. The first enantioselective synthesis of anticapsin was presented by Baldwin and co-workers^36^ (Scheme 3B). All other known methods of anticapsin synthesis can be found in the review paper on natural epoxycyclohexanes^37^.

Baldwin’s synthesis of anticapsin started with a chiral alcohol **1**, the preparation of which was designed by Kobayashi *et al.*^38^. The secondary hydroxyl group of this alcohol was transformed into the *tert*-butyldiphenylsilyl ether and then saponified using potassium trimethylsilanolate, to yield compound **2**. A three-step decarboxylation of **2** *via* thiohydroxamic ester afforded bis-silyl ether **3**, which was then gradually transformed into primary iodide **4.** Alkylation of this halide with **5** led to the formation of compound **3** which was subsequently hydrolysed to enantiomerically pure amino acid derivative **7**. Next, two selective oxidation reactions were performed, to obtain derivative **8** which was treated with specific enzymes to yield a free anticapsin ^36^.

**Scheme 4.**
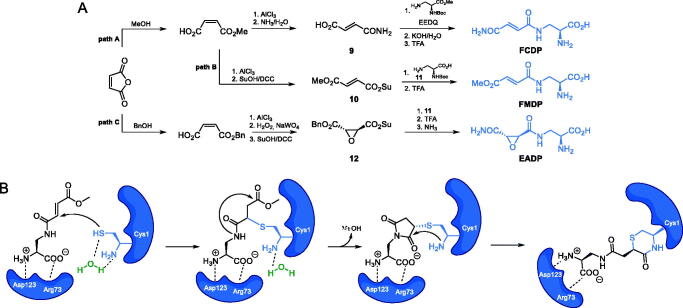
(A) Synthesis of GlcN-6-P synthase inhibitors containing l-2,3-diaminopropanoic moiety. (B) Molecular mechanism of GlcN-6-P synthase inactivation at GAH by FMDP^43^.

During the screening program of *Streptomyces*, Molloy et al. isolated compound **A190106** that exhibited growth inhibitory activity on *Salmonella gallinarum* (MIC = 8 μg/ml)^39^. Structural analysis of this metabolite revealed that it was a dipeptide containing a fumaramic acid moiety (Scheme 3A). Van der Baan et al.^40^ synthesised the N-terminal amino acid of this dipeptide, **FCDP**, using the route shown in Scheme 4A, path A. The authors used fumaric acid, which can be easily obtained from maleic anhydride, as a starting material, transformed into amide **9** *via* ammonolysis and coupled it with a methyl ester of *N*-α-*tert*-butoxycarbonyl-l-2,3-diaminopropanoic acid, using EEDQ as a coupling agent. The formed product was then hydrolysed with aqueous KOH and then with TFA, what led to the eventual formation of **FCDP**^40^.

Chmara *et al*. suggested that the biological activity of FCDP-Ala can be attributed to the inactivation of GlcN-6-P synthase by **FCDP** (*K*_i_ = 85 µM)^41^. After discovering of inhibitory activity of **FCDP** on *Salomonella typhimurium* GlcN-6-P synthase, Andruszkiewicz *et al*. synthesised FCDP analogues. In this series of compounds, one of them, namely **FMDP**, demonstrated significantly higher inhibitory potential against bacterial and yeast GlcN-6-P synthase (IC_50_ = 15–21 µM) than **FCDP** (82–100 µM) and inactivated the *Candida albicans* enzyme with *K*_i_ = 0.1 µM^42^. To obtain this compound, the authors converted maleic anhydride into an active ester of monomethyl fumarate **10**, coupled it with the terminal amino group of *N*-α-tert-butoxycarbonyl-L-2,3-diaminopropanoic acid **11** and then hydrolysed with trifluoroacetic acid, to obtain **FMDP** (Scheme 4A, path B)^42^. Kucharczyk et al. found that **FMDP** formed a covalent bond with the Cys1 residue of bacterial GlcN-6-P synthase, upon Michael-type nucleophilic addition of -SH functionality to the conjugated double bond system of the inhibitor molecule, which is followed by the formation of the 1,4-thiazin-3-one derivative, containing the substantial part of the Cys1 backbone^43^, as shown in Scheme 4B.

In the following works, Andruszkiewicz and co-workers tested the inhibitory activity of epoxysuccinic derivatives of 2,3-diaminopropanoic acid and found **EADP** (Scheme 4A, path C), along with some of its enantiomers and diastereoisomers, to be competitive inhibitors of GlcN-6-P synthase from *Saccharomyces cerevisae* (for **EADP**, *K*_i_ = 40 µM)^44^. **EADP** was synthesised starting from maleic anhydride, which was transformed into methyl fumarate, as shown previously and then its C = C bond was oxidised to epoxide **12** which was subsequently coupled with **11** and then subjected to a two-step deprotection process (Scheme 4A, path C)^44^.

**Scheme 5.**
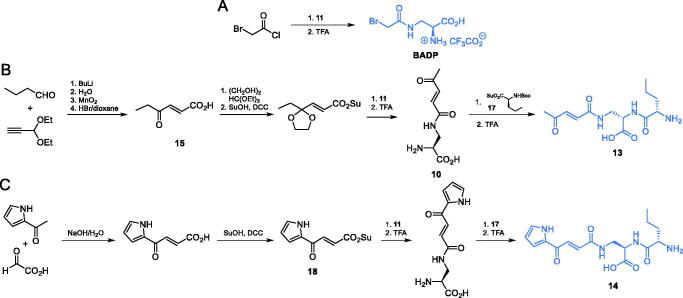
Synthesis of GlcN-6-P synthase inhibitors containing l-2,3-diaminopropanoic moiety.

To explore the topology of the glutamine-binding site of GlcN-6-P synthase, Auvin et al. obtained *N*-ω-haloacetyl derivatives of α,ω-diaminoalkanoic acids, out of which Nω–bromoacetyl-L-2,3-diaminopropionic acid **(BADP)** (Scheme 5A) showed parameters of GlcN-6-P synthase inactivation comparable to those of **FMDP** (*K*_i_ = 0.1 µM)^45^. To obtain that inhibitor, the authors used the Boc-protected l-2,3-diaminopropionic acid **11** which was acetylated with bromoacetyl chloride. Deprotection of the intermediate led to the final product in the form of a TFA salt (Scheme 5A)^45^.

Another series of l-2,3-diaminopropanoic acid derivatives that exhibit GlcN-6-P synthase inhibiting properties, including compounds **13** and **14** (Scheme 5B and C) were obtained by Walkowiak et al.^46^ The antifungal activity of these compounds was poor, with MIC values in the 62.5–125 µg/mL range. To obtain compound **13**, the authors utilised a condensation reaction between butanal and propionaldehyde diethyl acetate, followed by oxidation and hydrolysis, to obtain ketone **15** (Scheme 5B). The carbonyl group of this ketone intermediate was protected *via* acetal formation and then treated with TFA to obtain compound **16**, which was subsequently coupled with appropriately protected L-norvaline derivative **17** and once again treated with TFA, to finally yield dipeptide **13.** On the other hand, compound **14** was prepared *via* an aldol condensation of 2-acetylpyrrole and 2-oxoacetic acid, which yielded pseudofumarate **18** (Scheme 5C). Carboxylic acid activation using DCC/HOBt and consecutive conjugation of the protected amino acid was performed similarly as in the B route and led to the ultimate formation of **14**^46^.

It is noteworthy, that all L-2,3-diaminopropanoic acid-based inhibitors of GlcN-6-P synthase exhibit high selectivity towards this amidotransferase^47^. Such a feature is unique because other glutamine analogs, like 6-diazo-5-oxo-l-norleucine (DON) or azaserine are inhibitors of many glutamine-utilising enzymes^48^. Interestingly, anticapsin, not based on the l-2,3-diaminopropanoic acid scaffold, is also a selective inhibitor of GlcN-6-P synthase.

**Scheme 6.**
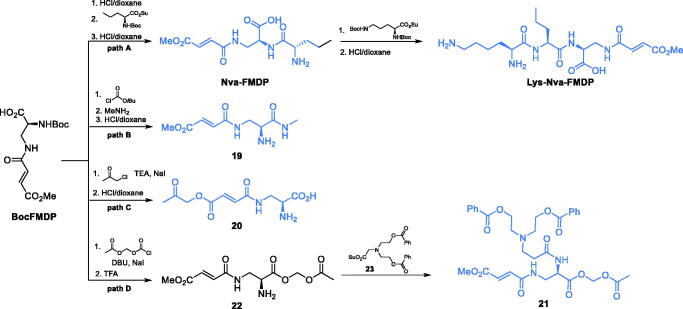
Synthesis of antimicrobials incorporating FMDP.

Due to the high affinity of **FMDP** to GlcN-6-P synthase, this compound was selected as a leader in the search for antimicrobial agents targeting that enzyme. Unfortunately, its intrinsic antimicrobial activity was low (MICs in the 100–200 µg/mL range). A substantial improvement was achieved upon the incorporation of **FMDP** into oligopeptide structures, with two compounds, namely **Nva-FMDP** and **Nva-Lys-FMDP**, designed and obtained by Andruszkiewicz *et al*. (Scheme 6, path A)^49^. Those compounds exhibited the highest *in vitro* and *in vivo* activity against *C. albicans* and several other human pathogenic fungi^50^ but were also active against bacteria^51^. The FMDP-oligopeptides are transported into microbial cells by oligopeptide permeases and then cleaved by intracellular peptidases^50^^,^^52^. Recently, Nva-FMDP was found to exhibit promising growth inhibitory activity against Fluconazole-resistant cells of the emerging fungal pathogen *Candida auris*^53^.

Conversion of **FMDP** into its more lipophilic derivatives was another way of potentiation of its antimicrobial activity. Zgódka *et al*. synthesised the acetoxymethyl ester of **FMDP**, which demonstrated about 20 times lower MIC value against *C. albicans* than the parent amino acid^54^. Pawlak et al. designed another series of compounds, trying to take advantage of the antimicrobial potential of **FMDP**. The authors focussed their attention on the alpha-carboxyl group of **FMDP** as a suitable site of the structural modification and transformed it into amides, one of which (**19**) is shown on Scheme 6, path B^55^. To obtain that compound, the authors used *N*-Boc-FMDP as a precursor and transformed it with isobutyl chloroformate into a mixed anhydride, which was subsequently aminolysis with methylamine and treated with dry HCl, to yield the final compound^55^. That FMDP amide showed some *in vitro* antifungal activity, however, it was limited exclusively to *C. albicans.* The same team also obtained esters and α-hydroxy ketones of **FMDP** (Scheme 6, path C). In that case, *N*-Boc-FMDP was also used as starting point in the synthesis. The 2-oxoalkoxyl function was introduced by the reaction of BocFMDP with chloroacetone. Subsequent deprotection led to the formation of an ester **20**. That compound showed some moderate antifungal *in vitro* activity against *C. albicans* and *Candida glabrata*^56^. Koszel et al.^57^ synthesised derivatives of bicine conjugated with **FMDP**. Most of the synthesised compounds showed moderate antifungal activity, while retaining a good water solubility. The most potent compound **21** (Scheme 6, path D) was obtained from **BocFMDP**. The starting compound was treated with ((chlorocarbonyl)oxy)methyl acetate in the presence of DBU and sodium iodide. After deprotection of intermediate **22** with trifluoroacetic acid, the amino function was coupled with bicine derivative **23,** which led to the formation of **21**^57^.

**Scheme 7.**
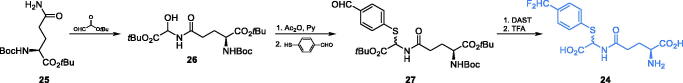
Synthesis of the mechanism-based GlcN-6-P synthase inhibitor, according to Massiere *et al*.^58^

Prompted by the need to find more specific inhibitors that target the glutamine-binding site of GlcN-6-P synthase, Massiere et al. designed compound **24** (Scheme 7)^58^. The authors proposed a mechanism of action of this inhibitor, in which after binding at the GAH active site it is decomposed to products that spontaneously generate powerful electrophilic species reacting with the nucleophilic residues at the GAH enzyme and in consequence, inactivating the enzyme. However, in practice, this potential mechanism-based inhibitor exhibited only moderate enzyme-inhibiting properties (*K*_i_ = 36 mM). That inhibitor was synthesised using the suitably protected glutamine which reacted with *tert*-butyl glyoxalate to give a glycine derivative **25**. The formed compound **26** was then acetylated and treated with 4-mercaptobenzaldehyde, which led to the formation of **27.** The aromatic group of this compound was then converted into difluoromethyl group. One-step de-protection of amino and carboxyl function led to the final compound **24** (Scheme 7)^58^.

**Scheme 8.**
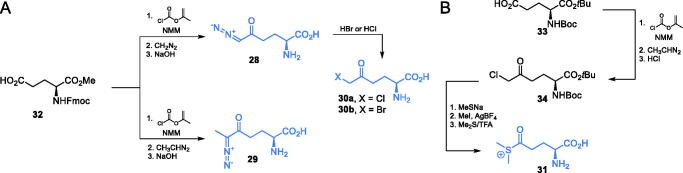
Synthesis of electrophilic glutamine-based inhibitors of GlcN-6-P synthase.

A series of other electrophilic glutamine analogues targeting GlcN-6-P synthase were reported in the literature. Walker *et al*.^59^ described irreversible inhibition of GlcN-6-P synthase by diazoalkyl derivatives: **28** (DON) and **29**, halometyl derivatives **30a-b** (Scheme 8A) and dimethyl sulfonium ketone (DSOK) **31** (Scheme 8B). To obtain diazoalkyl glutamine analogues **28** and **29**, the authors used the appropriately protected glutamic acid derivative **32** and reacted it with *in situ* generated *N*-nitroso-β-isobutylalkyl ketone, which led to the formation of appropriate diazoalkyl ketone. Compound **28** was also used as a precursor for compounds **30a-b**. By treating diazoketone **28** with HBr or HCl, compounds **30a** or **30 b** were obtained respectively. To obtain DSOK **31**, a protected glutamate **33** was transformed into respective chloromethyl ketone **34**. This intermediate compound was transformed into methyl sulfide with sodium methanethiolate and then methylated with methyl iodide to form a dimethylsulfonium ion. Deprotection with trifluoroacetic acid led to compound **31.** DSOK (**31**) appeared one of the strongest inactivators of GlcN-6-P synthase (*K*_i_ = 0.37 µM)^59^.

Several other glutamine analogues, like 6-diazo-5-oxo-L-norleucine, azaserine or γ-glutamate semialdehyde^60^ are effective inhibitors of GlcN-6-P synthase^18^ but for the lack of enzyme inhibitory selectivity, they cannot be considered promising drug candidates.

## 4. Fructose-6-phosphate and glucosamine-6-phosphate analogues

**Scheme 9.**
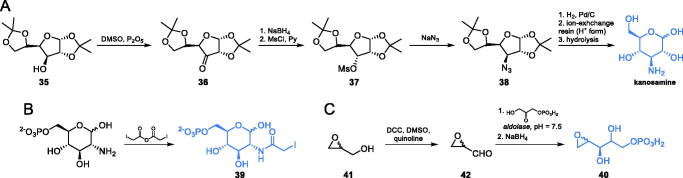
Syntheses of GlcN-6-P and Fru-6-P analogues targeting GlcN-6-P synthase.

Kanosamine, i.e. 3-amino-3-deoxy-d-glucose, is an antibiotic substance produced by *Bacillus aminoglucosidicus*. Mechanism of its antifungal action comprises uptake by the glucose transport system, intracellular conversion into kanosamine-6-phosphate and inhibition of GlcN-6-P synthase by this derivative, competitive in regard to Fru-6-P, with *K*_i_ = 5.9 mM^61^. The synthesis of kanosamine was reported by Meyer zu Reckendorf et al. (Scheme 9A). In the presented approach, the authors used the sugar derivative **35** which was oxidised to ketone **36.** The formed ketone was then stereoselectively reduced with sodium borohydride and transformed into a corresponding mesylate **37**, which was subsequently reacted with sodium azide *via* S_N_2 reaction, to obtain azide **38**. Catalytic hydrogenation of **38**, followed by ion-exchange resin-mediated hydrolysis, led to *N-*acetylkanosamine which can be hydrolysed to the free amino sugar^62^.

The first irreversible inhibitor of GlcN-6-P synthase targeting the ISOM domain was described by Bearne et al.^63^ In their studies, they obtained *N*-iodoacetylglucosamine-6-phosphate **39**, designed as a structure that mimics the final product formed at the active centre of this domain. Compound **39** was obtained through the reaction of commercially available glucosamine-6-phosphate and iodoacetic anhydride (Scheme 9B). The authors strictly controlled the pH of the reaction, which led to the formation of the final product with a high yield. Compound **39** inactivated GlcN-6-P synthase with *K*_i_ = 0.22 mM^63^.

Leriche et al.^64^ studied the Fru-6-P binding site at the ISOM domain of *E. coli* GlcN-6-P synthase, using anhydro-1,2-hexitol-6-phosphate **40** (Scheme 9C), a structural analogue of an open ring form of Fru-6-P, which was previously identified as an irreversible inhibitor of phosphoglucose isomerase. To obtain **40**, the authors used racemic glycidol **41** as a starting material, which was oxidised to a corresponding aldehyde **42** *via* the Moffat oxidation. The formed aldehyde was then chemoenzymatically condensed with glycerone phosphate, using aldolase. The resulting intermediate was then reduced using sodium borohydride which led to the racemic **40.** This compound inactivated GlcN-6-P synthase, with *K*_i_ = 1.4 mM^64^.

## 5. Analogues of transition state intermediates at the ISOM active site

**Scheme 10.**
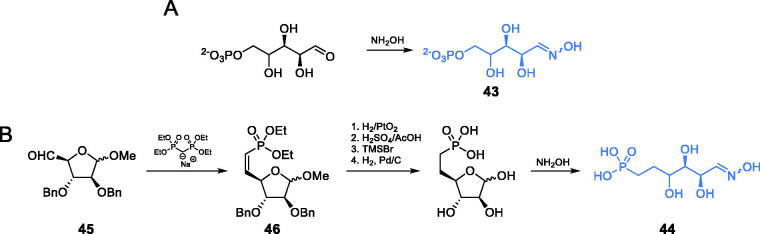
Synthesis of fructosimine-6-P analogues.

Le Camus and co-workers found arabinose-5-phosphate oxime **43** (APO) to be a potent inhibitor of GlcN-6-P synthase^65^. The authors obtained the aforementioned compound by converting the commercially available arabinose-5-phosphate to its oxime with hydroxylamine (Scheme 10A). Due to the low hydrolytic stability of phosphate moiety in **43**, the authors decided to obtain its homolog **44**. This compound was obtained using d-arabinose-derived aldehyde **45**, which was converted into vinylphosphonate **46** using the Horner-Emmons reaction. A Series of selective deprotection reactions, followed by a reaction with hydroxylamine, led to the final formation of oxime **44** (Scheme 10B). Both compounds, **43** and **44**, can be considered as structural analogues of open ring fructosamine-6-P, formed at the ISOM active site from Fru-6-P after its amination with glutamine-derived ammonia. The enzyme inhibitory potential of **43** was quite high (*K*_i_ = 14.3 µM), while that of **44** was much lower (*K*_i_ = 0.36 mM)^65^.

**Scheme 11.**
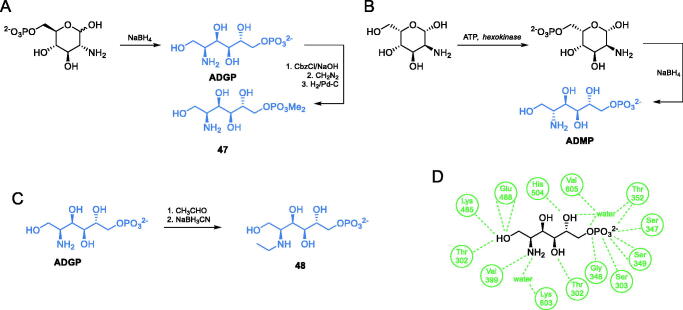
(A–C) Synthesis of transition state *cis*-enolamine analogues. (D) ADGP binding at *E. coli* GlcN-6-P synthase ISOM active site (based on pdbid: 1mos); H-bonds are shown by dashed lines.

Investigating the Fru-6-P binding site of *E. coli* GlcN-6-P synthase, Badet-Denisot and co-workers synthesised 2-amino-2-deoxyglucitol-6-phosphate (**ADGP,** Scheme 11A), which turned out to be a strong inhibitor of the enzyme, with *K*_i_ = 25 µM^66^. This compound is considered a structural mimic of *cis*-enamine intermediate. The authors obtained **ADGP** using a standard reduction of GlcN-6-P with sodium borohydride. The antimicrobial activity of **ADGP** is low (MIC = 5 mg/mL), but its high enzyme inhibitory potential inspired Janiak et al. to obtain and determine the enzyme inhibitor activity of ADGP derivatives. While most of them were found to be poorer inhibitors of GlcN-6-P synthase than the parent compound, some of them presented better antifungal *in vitro* activity. The most active compound was the dimethyl ester of **ADGP − 47**, with MIC values in the 0.3–0.6 mg/mL range^67^. This compound was obtained by the transformation of **ADGP** to its *N*-benzyloxycarbonyl derivative, followed by phosphate methylation using diazomethane. Subsequent deprotection of amino function by standard hydrogenation on Pd/C, led to the formation of **47**^67^. In continuation of this study, Milewski et al. reported that 2-amino-2-deoxy-d-mannitol-6-phosphate (**ADMP,** Scheme 10B) was found to be another potent inhibitor of GlcN-6-P synthase, actually stronger than **ADGP**^68^. That inhibitor was prepared using a commercially available 2-amino-2-deoxy-d-mannose *via* chemoenzymatic phosphorylation and subsequent reduction with sodium borohydride (Scheme 10B)^68^. Melcer et al. studied *N-*alkyl and *N,N*-dialkyl derivatives of **ADGP**, which exhibited higher antifungal activity than the parent compound, due to the better uptake by fungal cells. The most potent inhibitor **48** (Scheme 11B) was prepared by subsequent exhaustive reductive amination with acetaldehyde^69^.

## 6. Heterocyclic inhibitors targeting the active sites of GlcN-6-P synthase

### 6.1. Inhibitors based on five-membered ring scaffolds

**Scheme 12.**
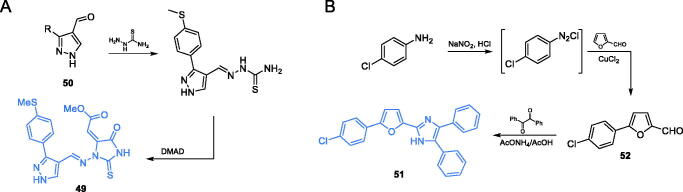
(A) Syntheses of putative GlcN-6-P synthase inhibitors, according to Vijesh *et al*.^70^ (B) Syntheses of presumable GlcN-6-P synthase inhibitors, according to Tomi *et al*.^72^

Vijesh et al.^70^ reported the synthesis of the imidazole derivatives containing imidazole-based scaffold, supposed to be potent antimicrobials. Derivative of 2-thiooxoimidazolidin-4-one imidazole **49** was obtained in excellent yield by refluxing 3-aryl-1*H*-pyrazole-4-carbaldehyde **50** with thiosemicarbazone in the presence of anhydrous sodium acetate and then refluxed with dimethylacetylenedicarboxylate (DMAD) (Scheme 12A)^71^. The *in vitro* antimicrobial activity studies showed that some of the trisubstituted imidazole derivatives exhibited growth inhibitory effect against tested microorganisms, with compound **49** emerging as the most active. Moreover, that compound exhibited higher activity against *Pseudomonas aeruginosa* than streptomycin. Molecular docking of **49** to the GlcN-6-P synthase matrix (PDB 2VF5) revealed that this ligand may bind to the active site of ISOM due to the interactions with Thr352 and Lys603, with estimated K_i_ of 8.56 µM^71^.

Some promising antimicrobial activity of triaryl-substituted imidazoles was reported by Tomi and co-workers^72^. In those studies, three imidazole derivatives were obtained and their antibacterial and antifungal activity was evaluated. Synthesis of compound **51** started with the conversion of *p*-chloroaniline to diazonium salt, followed by conjugation with furfural in the presence of CuCl_2_, thus giving the 5-substituted furfural **52**. Formation of the imidazole ring was accomplished by reaction of the obtained aldehyde with benzil and ammonium acetate under acidic conditions. As a result of condensation, the final derivative **51** was obtained (Scheme 12B)^72^. The studies on the antimicrobial potential of **51** showed that this compound exhibited the highest activity against Gram-negative bacteria (*E. coli, P. aeruginosa* and *Klebsiella pneumoniae*), actually higher than that of ampicillin. Moreover, results of molecular docking studies revealed that **51** may bind to the ISOM domain active site of GlcN-6-P synthase (PDB 1MOQ) by interaction with Gly301, with the estimated inhibition constant *K*_i_ = 2.59 µM and binding energy of −7.62 kcal/mol^72^.

**Scheme 13.**
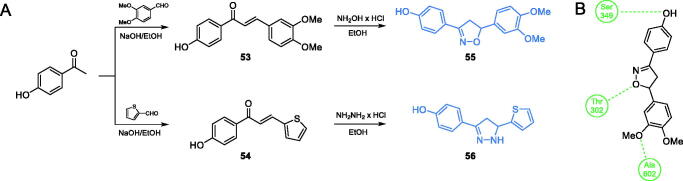
(A) Synthesis of disubstituted 1,2-oxazole and 1,2-diazole based possible inhibitors of GlcN-6-P synthase, according to Ismail *et al*.^73^ (B) Predicted binding mode of compound **55** at the ISOM active site; H-bonds are shown by dashed lines.

Ismail and co-workers^73^ characterised a series of 1,2-diazole- and 1,2-oxazole-based compounds as potential antimicrobial agents. The synthesis of inhibitors was a two-step procedure, in which *p-*hydroxy acetophenone was condensed in an aldol manner with 3,3-dimethoxybenzaldehyde or thiophene-2-carbaldehyde, resulting in chalcones **53** and **54**. Subsequently, chalcones served as substrates for cyclisation reactions with hydroxylamine or hydrazine hydrochloride that led to final oxazole- and diazole-based compounds **55** and **56**, respectively (Scheme 13)^73^. Derivatives **55** and **56** exhibited the highest antimicrobial potential in disc diffusion tests, actually higher than that of amoxicillin. The molecular docking studies of **55** and **56** to GlcN-6-P synthase (pdbid: 1moq) showed that obtained inhibitors may bind to the ISOM active site *via* interactions with Ala602, Ser349 and Thr302 residues. Estimated values of K_i_ for **55** and **56** were 0.769 and 4.21 µM and their binding energies were −8.34 and −7.33 kcal/mol, respectively^73^.

**Scheme 14.**
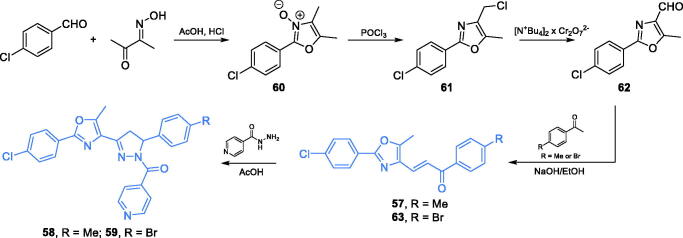
Synthesis of 1,3-oxazole- and 1,2-diazole-based putative inhibitors of GlcN-6-P synthase, according to Katariya *et al*.^74^

Antimicrobial agents based on 1,3-oxazole and 1,2-diazole scaffolds were synthesised by Katariya and co-workers^74^. The synthetic approach for **57**–**59** depended on the generation of the 1,3-oxazole ring of compound **62**. To accomplish the cyclisation, *p*-chlorobenzaldehyde and diacetyl monooxime were condensed, resulting in *N*-oxide **60**, which was converted to chloride **61** by treatment with POCl_3_. Subsequent oxidation with bis-tetrabutylammonium dichromate (bis-TBAC) resulted in aldehyde **62** that reacted in an aldol manner with appropriately substituted acetophenone, giving chalcone **63** and compound **57**. Finally, cyclisation of chalcones **57** and **63** with isoniazid in glacial acetic acid led to compounds **58** and **59** (Scheme 14)^74^. Compounds **57**–**59** exhibited the highest activity against bacterial and fungal cells, with MIC values as low as 6.25 µg/mL. *In silico* molecular docking studies accomplished on GlcN-6-P synthase (PDB 2VF5) revealed that **58** and **59** may interact with the ISOM domain active site *via* H-bonding with Thr302, Thr352, and Ser347 residues, while derivative **57** formed H-bonds with Thr352 and Asp354. The docking scores obtained for **57**–**59** were −52.263, −62.113, and −57.586, respectively^74^.

**Scheme 15.**
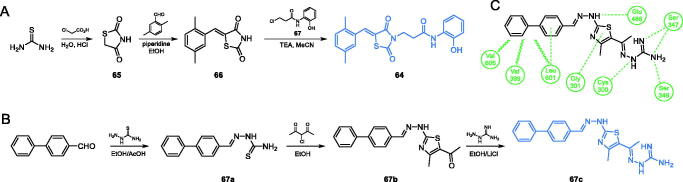
(A) Synthesis of a potential GlcN-6-P synthase inhibitor, according to Bahare *et al*.^75^ (B) Synthesis of GlcN-6-P synthase inhibitor, according to Omar et al.^76^ (C) Predicted binding mode of **67c** at the ISOM active site; H-bonds are shown by dashed lines; hydrophobic interactions are shown by wavy lines.

Bahare and co-workers described 2,4-thiazolidinedione-based compounds exhibiting significant antimicrobial activities^75^. The synthesis of **64** started with condensation of thiourea and chloroacetic acid, which led to 2,4-thiazolidinedione **65**, which underwent the aldol reaction with 2,5-dimethylbenzaldehyde, resulting in compound **66**. The final alkylation of the nitrogen atom with alkyl chloride **67**, gave **64** as a final compound (Scheme 15)^75^. *In vitro* evaluation accomplished on two fungal strains showed that compound **64** was a strong antifungal agent, with MIC values of 3.12 and 6.25 µg/mL against *C. albicans* and *Aspergillus niger*, respectively. It is noteworthy, that those values were lower than that for fluconazole (12.5 µg/mL). Further molecular docking studies were done on GlcN-6-P synthase (PDB 2VF5) revealed presumable three important H-bond interactions of **64** with Thr302, Val399 and Ala602 residues of the ISOM domain active site^75^.

Omar and co-workers^76^ reported the synthesis of a series of four compounds based on a thiazole scaffold. Obtained compounds possessed hydrophilic guanidine moiety as well as a lipophilic hydrocarbon-based substituent of aromatic nature, both responsible for interaction with particular residues at the ISOM. The synthesis of the most potent inhibitor **67c** (Scheme 15B) was accomplished in a three-step procedure starting from the condensation of a biphenyl aldehyde and a thiosemicarbazide in acidic conditions of acetic acid. The resulting carbothioamide **67a** was subsequently cyclized to thiazole derivative **67b** by reaction with 3-choloro-2,4-dioxopentane and finally guanidine residue was introduced by imine bond formation with aminoguanidine hydrochloride in the presence of LiCl. Antimicrobial evaluation of obtained compounds, accomplished on Gram-positive bacteria (*S. aureus* and *B. subtilis*) and fungi (*C. albicans* and *A. oryzae*) revealed that derivative **67c** was an excellent antibacterial agent, with MIC values comparable to those of ciprofloxacin (1 µg/mL for *S. aureus* and 0.5 µg/mL for *B.subtilis*). The antifungal activity of **67c** was even better and in the case of *C. albicans* MIC value was twice as better (4 µg/mL) when compared to standard fluconazole (8 µg/mL). Moreover, compound **67c** exhibited significant activity against the MRSA strain (MIC value 32 µg/mL) when compared to standard linezolid and vancomycin (1 µg/mL for both drugs). Under *in vitro* conditions, **67c** inhibited GlcN-6-P synthase activity with IC_50_ = 3.47 µM. Molecular docking studies performed on GlcN-6-P synthase matrix (pdbid: 2vf5) showed that **67c** interacts with the ISOM domain active site in the mode similar to that of GlcN-6-P and is stabilised by interactions with Val399, Ser347, Gln348, Glu488 (catalytic residue), Val605 and Leu601^76^, as shown in Scheme 15C.

**Scheme 16.**
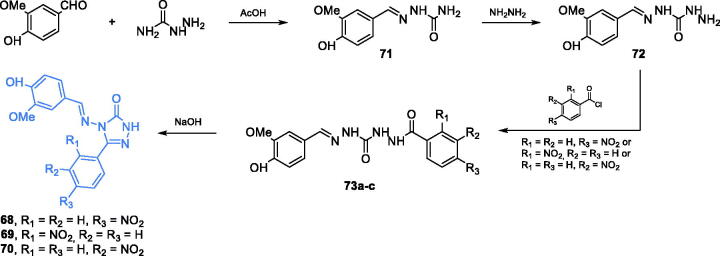
Synthesis of triazole-based putative inhibitors of GlcN-6-P synthase, according to Rajasekaran *et al*.^77^

Triazole derivatives **68–70** were synthesised by Rajasekaran and co-workers as potential GlcN-6-P synthase inhibitors. These compounds were obtained from 4-hydroxy-3-methoxybenzaldehyde and hydrazinecarboxamide, which were condensed under acidic conditions to semicarbazone **71**. A subsequent substitution of NH_2_ group with hydrazine resulted in derivative **72** that was acylated with appropriately substituted benzoyl chloride, resulting in **73a–c**. The final cyclisation, accomplished in basic conditions, gave the final compounds **68–70** (Scheme 16)^77^. The antimicrobial evaluation revealed that obtained compounds exhibited significant activity against Gram-negative and Gram-positive bacteria, with MIC values of 3.125–6.25 µg/mL. Good antifungal activity, especially against *C. albicans*, was observed for compounds **69** and **70**, with MIC values of 3.125–12.5 µg/mL. Results of the docking study of **68–70** performed on GlcN-6-P synthase matrix (pdbid: 1jxa) showed the putative high affinity of those derivatives to the active site of the ISOM domain and three common H-bond interactions with non-crucial Ser347, Thr352 and Val399 residues were identified. The docking scores obtained for **68–70** were −9.17, −9.98, and −8.98, respectively^77^.

**Scheme 17.**
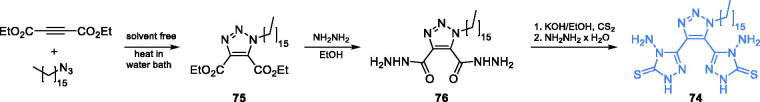
Synthesis of trisubstituted 1,2,3-triazole as a potential inhibitor of GlcN-6-P synthase, according to Aouad *et al*.^78^

Trisubstituted 1,2,3-triazole **74** synthesised by Aouad and co-workers has been recognised as a good inhibitor of microbial growth^78^. Click chemistry and solvent-free approach were applied for the synthesis of triazole intermediate **75** which was obtained by 1,3-dipolarcycloaddition of diethyl acetylenedicarboxylate and 1-azidopentadecane. The resulting heterocycle was subsequently aminolysis with hydrazine, thus giving **76**, which eventually was converted into the desired inhibitor in a two-step manner depending on treatment with carbon disulphide under basic conditions, followed by the addition of hydrazine (Scheme 17)^78^. The MIC values obtained against Gram-positive and Gram-negative bacteria ranged between 1 and 16 µg/mL, compared to 1–8 µg/mL obtained for ciprofloxacin. It is noteworthy, that in the case of *Bacillus cereus* and *P. aeruginosa,* the obtained MIC values were two times lower than that for the standard drug. Derivative **74** exhibited also good activity against fungal strains, especially *C. albicans*. The MIC value for this compound was the same as that for a standard antifungal, fluconazole (1 µg/mL). *In silico* studies on **74** docking to GlcN-6-P synthase (pdbid: 2vf5) revealed possible strong binding to the ISOM domain active site, with estimated *K*_i_ = 0.17 µM and binding energy of −9.23 kcal/mol^78^.

**Scheme 18.**
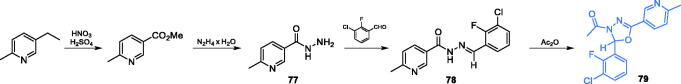
Synthesis of 1,3,4-oxadiazoles derivative as a potential inhibitor of GlcN-6-P synthase, according to Shyma *et al*. ^79^

Similarly to compounds based on imidazole or pyrazole rings, the 1,3,4-oxadiazole-based systems also exhibited significant biological activities, presumed to be due to the GlcN-6-P synthase inhibition^79^. Shyma *et al*. reported synthesis, characterisation and biological studies of 1,3,4-oxadiazoles containing 6-methyl pyridine moiety, as a continuation of their search for biologically potent molecules^80^^,^^81^. Starting from the 2-methyl 5-ethyl pyridine, the methyl 6-methyl nicotinate was prepared *via* oxidation using a nitrating mixture. This compound was treated with hydrazine hydrate to obtain carbohydrazide **77,** which subsequently reacted with 3-chloro-2-fluorobenzaldehyde, giving the corresponding hydrazone **78**. The final step, leading to the target inhibitor **79** involved 1,3,4-oxadiazole ring formation, which was accomplished by cyclisation of **78** in the presence of acetic anhydride (Scheme 18)^79^. The obtained product was screened for antibacterial and antifungal properties. Compound **79** emerged as most active against all tested bacterial strains, with growth inhibitory potential similar to that of the standard drug, streptomycin. Moreover, some antifungal activity of compound **79** was also found, although lower than that of the standard drug fluconazole. As revealed in the molecular docking studies, compound **79** should bind to the active pocket of GlcN-6-P synthase (pdbid: 2vf5) at the ISOM domain with *K*_i_ = 2.24 µM^79^.

**Scheme 19.**
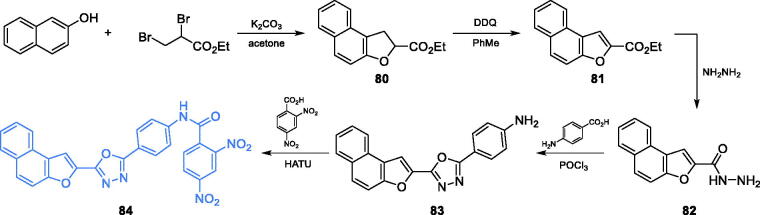
Synthesis of 2,5-disubstituted oxadiazole derivative as a potential inhibitor of GlcN-6-P synthase, according to Sindhe *et al*.^82^

A series of 2,5-disubstituted-1,3,4-oxadiazole agents exhibiting significant antimicrobial activity were obtained by Sindhe and co-workers^82^. The synthesis started from naphthofuran-2-hydrazide **82**. This compound could be obtained with good yields from 2-naphtol and ethyl 2,3-dibromopropionate, which underwent condensation and subsequent cyclisation to derivative **80**. Aromatisation of **80** with DDQ resulted in aromatic ethyl ester **81**^83^ that could be aminolysed with hydrazine to naphthofuran-2-hydrazide **82**. The cyclisation of **82** with *p*-aminobenzoic acid gave the new aromatic ring of oxadziazole **83**, which was eventually condensed with 2,4-dinitrobenzoic acid, using HATU as a coupling reagent, giving the final compound **84** (Scheme 19)^82^. Oxadiazole **84** exhibited moderate antimicrobial activity, with MICs ranging between 0.2 and 0.4 mg/mL, as compared to that of standard drugs, ciprofloxacin (0.2 mg/mL) against bacterial strains and fluconazole (0.2 mg/mL) against fungal strains. *In silico* molecular docking experiments performed on GlcN-6-P synthase (pdbid: 2vf5) revealed that **84** presumably interacts *via* five hydrogen bonds with non-crucial Val399, Ser303, Ser349, Thr302 and Ser401 residues at the ISOM domain active site, with the calculated binding energy of −9.3 kcal/mol^82^.

### 6.2. Inhibitors based on six-membered ring scaffolds

**Scheme 20.**
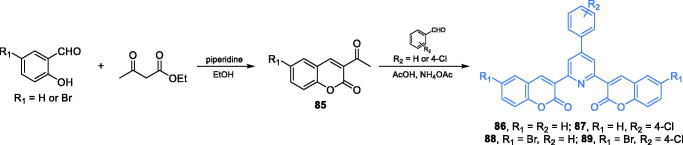
Synthesis of 2,4,6-trisubstituted pyridine-based putative inhibitors of GlcN-6-P synthase, according to Kenchappa *et al*.^84^

Some 2,6-bis(1-coumarin-2yl)-4–(4-substitutedphenyl)pyridine derivatives exhibiting moderate to good antimicrobial activity were reported by Kenchappa and co-workers^84^. The synthesis of compounds **86–89** started from the preparation of appropriately substituted coumarin **85**, which was obtained according to Vijesh et al.^85^, who condensed 2-hydroxybenzaldehyde or 5-bromo-2-hydroxybenzaldehyde with ethyl acetoacetate. Subsequently, the reaction of **85** with appropriate benzaldehyde derivative and ammonium acetate was accomplished leading to the formation of the pyridine ring of final products **86–89** (Scheme 20)^84^. The obtained compounds exhibited remarkable antibacterial activity, with MIC values comparable to those found for amoxicillin and gentamicin. Moreover, docking studies on GlcN-6-P synthase (pdbid: 2vf5) revealed the ability of those agents to bind to the ISOM domain active site *via* H-bonding with Gln348 and catalytic Lys603 residues. Estimated inhibition constants for **86–89** were 193.05, 226.14, 281.02, and 367.79 µM, respectively. Their binding energies ranged between −5.07 for **86** and −4.69 kJ/mol for **89**^84^.

**Scheme 21.**
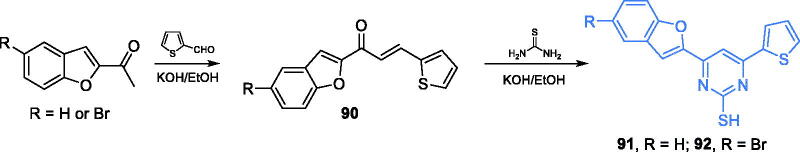
Synthesis of trisubstituted pyrimidine-based putative inhibitors of GlcN-6-P synthase, according to Venkatesh *et al*.^86^

Pyrimidine-based trisubstituted derivatives with good antibacterial and antifungal activity were synthesised and biologically evaluated by Venkatesh and co-workers^86^. The inhibitors were prepared in two-step synthesis depending on the formation of chalcone **90** by aldol condensation of appropriately substituted 2-acetylbenzofuran with thiophene carbaldehyde, followed by cyclisation reaction with thiourea (Scheme 21)^86^. Compounds **91** and **92** exhibited MICs in the 12–18 µg/mL range, in comparison to 17–19 µg/mL found for streptomycin. Evaluation of antifungal potency revealed MIC values in the 10–15 µg/mL range, compared to 17–18.5 µg/mL for fluconazole. Derivatives **91** and **92** showed the lowest binding energy (−5.77 and −5.29 kcal/mol) to the GAH domain active site (pdbid: 1xff) and ability to H-bonding with catalytic Cys1 and Trp74 residues, thus suggesting their potential as relatively good inhibitors of this enzyme^86^.

**Scheme 22.**
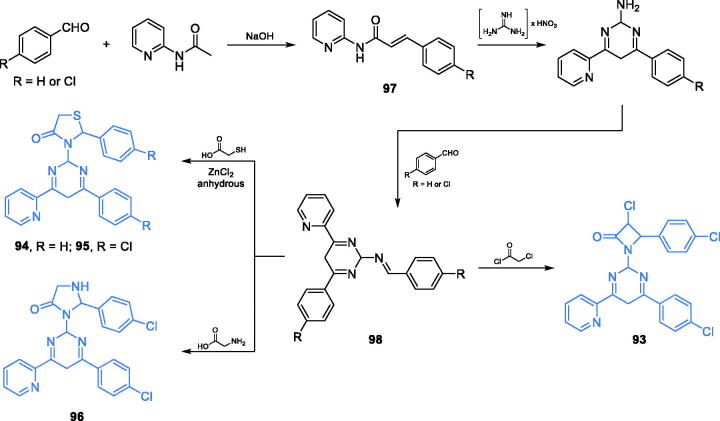
Synthesis of 2,4,6-trisubstituted 1,3-diazine-based potential inhibitors of GlcN-6-P synthase, according to Bakr *et al*.^87^

2,4,6-trisubstituted-1,3-diazines **93–96** (Scheme 22) were reported by Bakr and co-workers as promising antimicrobial agents^87^. Synthesis of these compounds started from condensation of an appropriately *p*-substituted benzaldehyde and 2-(acetylamino)pyridine that gave the α,β-unsaturated product **97**. The cyclisation accomplished with **97** and guanidine nitrate gave diazine scaffold, which was subsequently subjected to reaction with benzaldehyde derivative, thus leading to imine **98**. The imine was the starting material for three different cyclisation reactions. Condensation of **98** with 1-chloroacetic acid chloride resulted in the formation of an azetidine-2-on ring of inhibitor **93**, while reactions with 1-mercaptoacetic acid and glycine gave five-membered rings of inhibitors **94–96**, respectively (Scheme 22)^87^. Derivative **93**, possessing azetidine-2-one substituent, occurred to be the most potent antibacterial agent. On the other hand, the highest antifungal activity, comparable to that of amphotericin B, was observed for compounds **94**–**96** bearing thiazolidine-4-one and imidazoline-4-one substituents. Molecular docking studies based on the GAH domain of GlcN-6-P synthase (pdbid: 1xff) revealed the possible formation of H-bonds with Gly99 and arene-arene interaction with Trp74 located at the active centre. The binding energies for **93**, **95**, and **96** were −13.06, −16.89, and −15.76 kcal/mol, respectively^87^.

**Scheme 23.**
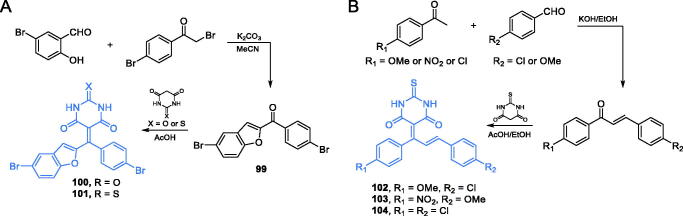
Synthesis of barbiturate- and thiobarbiturate-based possible GlcN-6-P synthase inhibitors, according to Kenchappa *et al*.^88^^,^^90^

A series of barbiturate- and thiobarbiturate-based derivatives containing benzofuran moieties were obtained and characterised by Kenchappa and co-workers^88^. The synthesis of two compounds (**100** and **101**) was accomplished by Knoevenagel condensation of appropriate methanone derivative **99** with barbituric or thiobarbituric acid (Scheme 23A)^88^. It is noteworthy, that the structural analogues of **100** and **101**, incorporating Meldrum’s acid, instead of barbitone moiety, were also synthesised and biologically evaluated by the research group of Kenchappa^89^. Compounds **100** and **101** displayed good antibacterial and antifungal activity, with MIC values ranging from 12.5 to 32 µmol/L. Further molecular docking studies on GlcN-6-P synthase (pdbid: 2vf5) revealed the protein-ligand interaction, depending on H-bond formation, with amino acid residues at the ISOM domain active site. In that simulations, compound **101** was found to interact with Gln348, Ser349, and Thr352 residues, with *K*_i_ = 280.61 µM and binding energy of −4.85 kJ/mol, while **100**, exhibiting estimated *K*_i_ = 229.07 µM and binding energy of −5.27 kJ/mol), formed H-bonds with Gln348 and Thr352 residues^89^.

Kenchappa *et al*. obtained also some chalcone derivatives of thiobarbiturates^90^. These compounds were obtained analogously to the previous ones, using the Knovenagel condensation (Scheme 23B)^90^. The biological evaluation of compounds **102–104** demonstrated some antimicrobial activity. Their MIC values for antibacterial action ranged from 28 to 37 µg/mL, compared to 25–27 µg/mL of the standard streptomycin. In the case of antifungal evaluation, MIC values of 23–42 µg/mL against three fungal strains have been obtained, in comparison to 20–24 µg/mL found for griseofulvin as a standard antifungal drug. *In silico* experiments accomplished on GlcN-6-P synthase (pdbid: 2vf5) exhibited that molecules **102** and **104** were supposed to form hydrogen bonds with Gln348 and Ser349 residues at the active site of the ISOM domain. The inhibition constants for **102** and **104** were estimated as 589.5 and 487.8 µM, respectively, and their binding energies were −4.41 and −4.52 kJ/mol^90^.

### 6.3. Inhibitors based on indene scaffolds

**Scheme 24.**
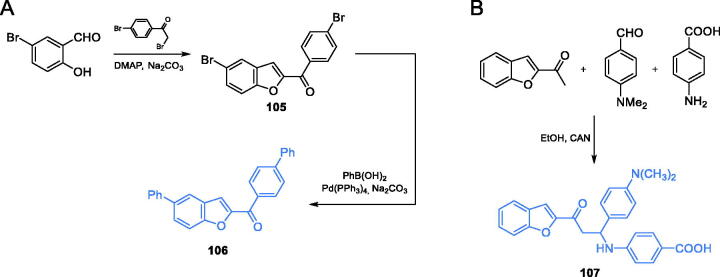
(A) Synthesis of benzofuran-2-yl derivative-based potential inhibitor of GlcN-6-P synthase, according to Aswathanarayanappa *et al*.^91^ (B) Synthesis of β-amino carbonyl derivatives of benzofuran as potential GlcN-6-P synthase inhibitors, according to Kenchappa *et al*.^92^

Aswathanarayanappa *et al*. proposed the synthesis of substituted 5-phenyl-1-benzofuran-2-yl derivatives^91^. The starting material was a benzofuran derivative **105**, which was prepared from 2-bromo-1–(4-bromophenyl)ethanone and 5-bromo-2-hydroxybenzaldehyde. Under basic conditions, a nucleophilic attack occurred on the aldehyde moiety, followed by cyclisation to form a benzofuran ring. The resulting compound **105** was subsequently treated with a boron compound in the presence of a tetrakis-(triphenylphosphine) palladium catalyst. Substitution of bromide atoms by introduced aryl or alkyl residues in aromatic rings was obtained *via* Suzuki coupling reaction, resulting in inhibitor **106** (Scheme 24A). Compound **106** appeared to be the most promising antimicrobial agent, thus it was active against *Staphylococcus aureus* (MIC of 1 µg/mL), *B. subtilis* (MIC of 10 µg/mL) and *C. albicans* (MIC of 10 µg/mL). Molecular docking studies (pdbid: 2vf5) showed possible binding of **106** to the active site of GlcN-6-P synthase ISOM domain, with an inhibition constant of 8.09 µM^91^.

Other benzofuran-derived compounds were proposed by Kenchappa and co-workers^92^. The synthesis was based on the known method developed by Vijesh *et al*.^85^ Compound **107** was obtained by the one-pot, three-component reaction between *p*-substituted benzaldehyde, *p-*substituted aromatic amine and 1–(1-benzofuran-2-yl) ethenone, in the presence of cerric ammonium nitrate (CAN) as a catalyst. CAN showed the best catalytic properties among all Lewis acids (ZnCl_2_, CuCl_2_, AlCl_3_, FeCl_3_, LaCl_3_, InCl_3_) proposed in this study. All products were obtained with good yields, on average 81% (Scheme 24B)^92^. The docking studies presented the benzofuran derivative **107** as a good inhibitor of GlcN-6-P synthase. That compound was docked to GlcN-6-P synthase (pdbid: 2vf5) matrix and showed to form hydrogen bonds with non-crucial Ser347 and Cys300 at the active site of the ISOM domain, with an inhibition constant of 22.19 µM^92^. The MIC values of **107** in the evaluation of its antimicrobial potential ranged between 40 and 50 µg/mL for all tested microorganisms (three bacterial and three fungal species), compared to those of the standard drugs, fluconazole (40 µg/mL) and streptomycin (30 µg/mL).

**Scheme 25.**
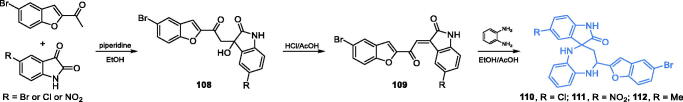
Synthesis of benzodiazepine-based potential inhibitors of GlcN-6-P synthase, according to Kenchappa *et al*.^93^

In another work, Kenchappa and co-workers characterised twelve spiro-derivatives of diazepine heterocycle, incorporating benzofuran- and indole-based moieties^93^. The proposed four-step synthesis started with an aldol reaction between 2-acetyl-5-bromobenzofuran and appropriately substituted isatin. Condensation product **108** was subsequently dehydrated under acidic conditions of HCl giving α,β-unsaturated derivative **109**. Eventual intramolecular cyclisation with four reaction centres took place between **109** and 2-aminoaniline and gave the final products **110–112** (Scheme 25)^93^. Compounds **110–112** displayed moderate antimicrobial activity when tested against four bacterial and four fungal strains. The results showed that heterocycles **110–112** were as good antibacterial agents as the standard drug ciprofloxacin and displayed MIC values ranging from 0.3 to 0.2 mg/mL. Antifungal activity of tested derivatives was similar to that of the standard fluconazole. The molecular docking of **110–112** to GlcN-6-P synthase (pdbid: 2vf5) demonstrated that those molecules interacted with residues Gly301, Cys300, Val399, and Ala602 of the ISOM domain active site. The estimated binding energies for **110** and **111** were −6.9 and −8.1 kcal/mol, respectively^93^.

**Scheme 26.**
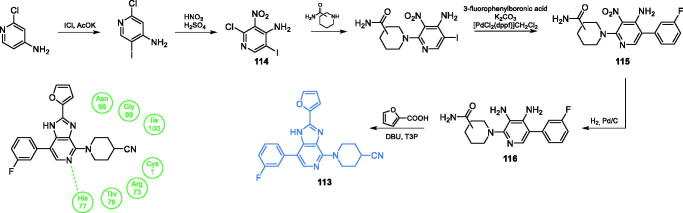
Synthesis of imidazo[4,5-C]pyridine-based potential GlcN-6-P synthase inhibitor, according to Jose *et al*.^94^ and its predicted binding mode to the GAH active centre of GlcN-6-P synthase; H-bonds are shown by dashed lines.

Novel imidazo[4,5-c]pyridine derivatives were synthesised and described by Jose *et al*.^94^ Synthesis of imidazo[4,5-C]pyridine derivative **113** started from the halogenation of 4-amino-2-chloro pyridine with ICl in the presence of glacial acetic acid and potassium acetate solution. From the resulting mixture of iodopyridines, 4-amino-2-chloro-5-iodo pyridine was separated. The subsequent nitration reaction resulted in pyridine derivative **114**, which was then treated with piperidine carboxamine, where the chlorine atom was substituted with the piperidine derivative. In the next step, the iodine group was replaced *via* Suzuki reaction in the presence of potassium carbonate as a required base and [PdCl_2_(dppf)]CH_2_Cl_2_ complex as a catalyst. The nitro group of compound **115** was reduced to diamine **116** and then cyclized to final product **113**. The final step was carried out in the presence of DBU and T3P under microwave irradiation with the use of furan-2-carboxylic acid, which caused the formation of an imidazole ring and introduced a furan-2-yl substituent (Scheme 26)^94^. Five out of twelve obtained imidazole derivatives showed moderate to good antimicrobial activity. Derivative **113** inhibited growth of Gram-negative bacteria *E. coli* (IC_50_ = 74.5 µg/mL) and that of *S. cerevisiae* yeast (IC_50_ = 99 µg/mL). Molecular docking to the GlcN-6-P synthase matrix (pdbid: 1jxa) showed the interaction between **113** as a ligand and His77 at the active site of the GAH domain, with *K*_i_ = 4.96 nM^94^, surprisingly low, assuming the only interaction with a single His residue.

**Scheme 27.**
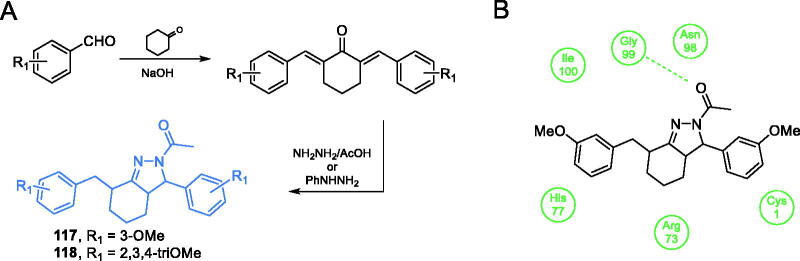
(A) Synthesis of curcumin derivatives as potential inhibitors of GlcN-6-P synthase, according to Kumar *et al*.^95^ (B) Predicted binding mode of compound **117** to the GAH domain of GlcN-6-P synthase; H-bonds are shown by dashed lines.

Kumar *et al*. proposed the synthesis of curcumin derivatives, antimicrobial properties of which were explained by their inhibitory action towards GlcN-6-P synthase. All compounds were obtained through Claisen-Schmidt condensation of substituted benzaldehydes with cyclohexanone in the presence of a base, followed by reflux with appropriate hydrazine derivatives (Scheme 27)^95^. Kumar’s work was based on previous results reported by Minu *et al*.^96^ about the synthesis of 2,3-disubstituted-3,3a,4,5,6,7-hexahydro-2*H*-indazole derivatives. Those authors observed enhanced biological activity of compounds that contained electron withdrawing groups, such as halogen groups, at the third position of hexahydroindazole. Kumar *et al*. analogously proposed a series of novel hexahydro indazole derivatives of curcumin with various substituents on the aryl ring. Antimicrobial activities of compounds **117** and **118** (Scheme 27), determined against Gram-positive and Gram-negative human pathogenic bacteria (*E*. *coli, P. aeruginosa, S. typhimurium* and *S. aureus*) and fungal microorganisms of the *Candida* spp. was promising. In molecular docking studies, both compounds are bound to the active site of the GAH domain. Compound **117** is supposed to interact with Gly99, with *K*_i_ = 0.045 µM and **118** interacted with Arg73 and His77, with *K*_i_ = 8.57 µM^95^.

**Scheme 28.**
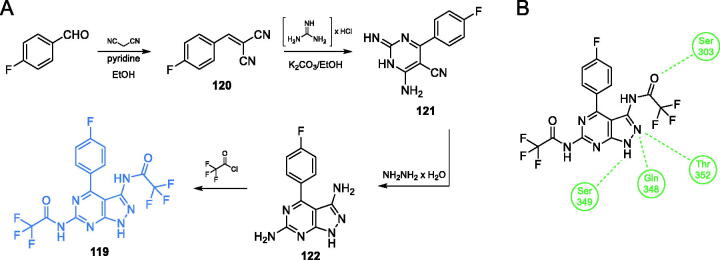
(A) Synthesis of fluorine-substituted pyrazolopyrimidine-based putative inhibitor of GlcN-6-P synthase, according to Khan *et al*.^97^ (B) Presumable binding mode of **119** to GlcN-6-P synthase at the ISOM active site; H-bonds are shown by dashed lines.

Khan *et al*.^97^ proposed compound **119** (Scheme 28), which exhibited good activity against *S. aureus*, *Streptococcus pyogenes*, *S. typhimurium*, and *E. coli*. In the synthetic approach for bicyclic aromatic system, the pyrimidine ring was obtained in the first step. To achieve that, *p*-fluorobenzaldehyde and malononitrile were condensed, resulting in alkene **120**, in which the nitrile moieties served as electrophiles in cyclisation reaction with guanidine hydrochloride, giving the heterocyclic ring of **121**. Subsequently, another cyclisation was performed, using hydrazine as a double nucleophile. The eventual acylation of primary amine groups of **122** with trifluoroacetic acid chloride resulted in the final inhibitor **119** (Scheme 28)^97^. The MIC values of this compound against bacteria ranged from 16 to 32 µg/mL when compared to that of the standard drug, chloramphenicol (32 µg/mL). Docking studies performed on bacterial GlcN-6-P synthase (pdbid: 2vf4) revealed the ability of **119** to bind to the ISOM domain active site by interaction with Ser303, Ser349, Gln348 and Thr352 residues, with the binding energy of −7.3 kcal/mol^97^.

**Scheme 29.**
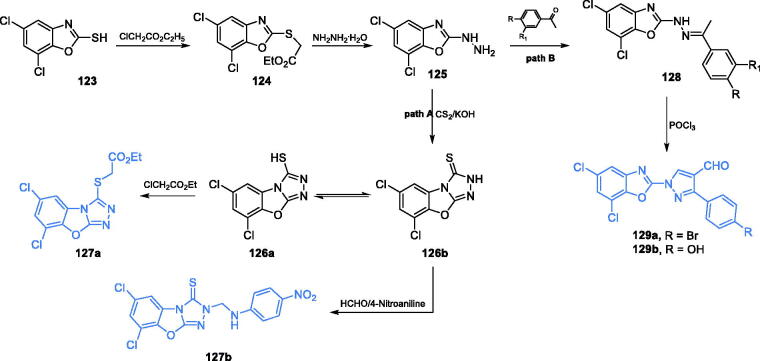
Synthesis of 5,7-dichloro-1,3-benzoxazole-2-thiol derivatives as potential inhibitors of GlcN-6-P synthase, according to Satyendra et al. (path A)^98^^,^^99^. Synthesis of 5,7-dichloro-1,3-benzoxazole-2-yl derivatives as potential inhibitors of GlcN-6-P synthase according to Jayanna et al. (path B)^100^.

Satyendra *et al*. described the synthesis, molecular docking studies and biological properties of 5,7-dichloro-1,3-benzoxazole-2-thiol derivatives^98^^,^^99^. The synthesis started with the formation of ethyl [(5,7-dichloro-1,3-benzoxazole-2-yl)sulfanyl]acetate **124** due to the reaction of commercially available 5,7-dichloro-1,3-benzoxazole-2-thiol **123** with ethyl chloroacetate (Scheme 29, path A). The resulting compound is treated with hydrazine to obtain 5,7-dichloro-2-hydrazino-1,3-benzoxazole **125**, which subsequently reacted with carbon disulphide in the presence of a strong base, resulting in compound **126**. Compound **126** has two tautomeric forms **126a** and **126b** and due to this property, two routes of further transformations were taken: acylation of a thiol group or substitution of the nitrogen atom in the newly obtained ring (Scheme 29, path A)^98^^,^^99^. All final compounds and some intermediates were screened for antimicrobial activity and potential inhibition of GlcN-6-P synthase. Compound **127a** emerged as the most active against all tested microorganisms and exhibited good antimicrobial activity, with MIC values of 3.125 µg/mL, compared to MIC of 3.125 µg/mL obtained for ciprofloxacin. Molecular docking to GlcN-6-P synthase (pdb: 1gdo) revealed that **127a** possibly binds at the active site of the GAH domain by interaction with Cys1 (*K*_i_ = 1.04 µM)^99^.

Benzoxazole ring was also an integral part of compounds, whose synthesis was proposed and described by Jayanna *et al*.^100^ Novel derivatives of benzoxazole were linked to pyrazole moiety containing an aldehyde group. The first step of synthesis was imine formation between 5,7-dichloro-2-hydrazino-1,3-benzoxazole **125** and appropriate 3,4-disubstituted acetophenone. The resulting compound **128** was subjected to a Vilsmeier-Haack reaction with POCl_3_ in DMF, to obtain five final products, including **129** (Scheme 29, path B)^100^. Compounds **129a** and **129 b** emerged as the most promising inhibitors of GlcN-6-P synthase, due to good antimicrobial activities (MIC values of 20–30 µg/mL for all strains for both compounds) and results of molecular docking to the target enzyme (pdbid: 2vf5). Compound **129a** formed hydrogen bonds with Thr352, with *K*_i_ = 280.61 µM and the binding energy of −4.85 kJ/mol, while **129 b** (binding energy of −8.19 kJ/mol) interacted with Thr352 and Glu488, at the active site of ISOM domain^100^.

**Scheme 30.**
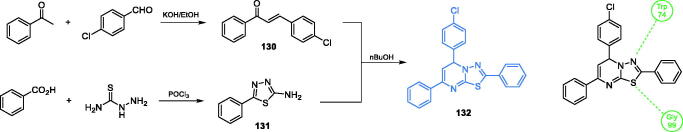
Synthesis of a potential GlcN-6-P synthase inhibitor, according to Venkatesh *et al*.^101^^,^^102^ and its predicted binding mode to the GAH domain; H-bonds are shown by dashed lines.

Efficient synthesis and promising antimicrobial activities of 5,7-disubstituted-2-phenyl-5*H*-[1,3,4]-thiadiazolo[3,2-*a*]pyrimidine derivatives were reported by Venkatesh and co-workers^101^. The synthesis of the final compounds called for the preparation of chalcone **130** and thiadiazole **131**^102^. The chalcone was obtained by an aldol reaction between acetophenone and *p*-chlorobenzaldehyde. The single-step reaction was also applied for the production of thiadiazole **131**, which was synthesised by condensation of benzoic acid and hydrazinecarbothioamide. Eventual conjugation of **130** and **131** connected with cyclisation reaction gave the final inhibitor **132** (Scheme 30)^101^^,^^102^. The most potent antimicrobial derivative **132** exhibited good activity against both bacterial and fungal cells. The MIC value in antibacterial tests ranged between 18 and 20 µg/mL, which was comparable to that of the standard drug, ciprofloxacin (20–23 µg/mL). Evaluation of the antifungal activity of **132** showed MIC values in the 25–28 µg/mL range, which was comparable to that of the standard fluconazole (30–32 µg/mL). Molecular docking simulations accomplished on the GAH domain of GlcN-6-P synthase (pdbid: 1xff) showed that derivative **132** could interact *via* H-bonding with Trp74 and Gly99 residues of the GAH domain active site, with the binding energy estimated as −10.1 kcal/mol^101^.

### 6.4. Inhibitors based on naphthalene-based scaffolds

**Scheme 31.**
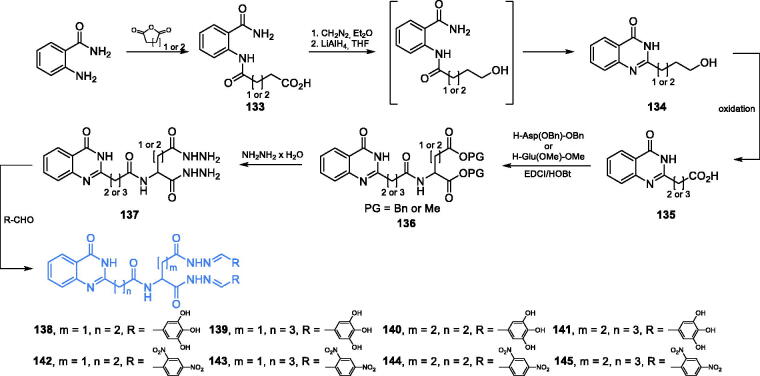
Synthesis of quinazolinone-based putative inhibitors of GlcN-6-P synthase, according to Kumara *et al*.^103^

The preparation of a series of eight potent antimicrobial agents based on a quinazolinone structure was reported by Kumara and co-workers^103^. The quinazolinone scaffold of the inhibitors was obtained from 2-aminobenzamide and succinic or glutaric anhydride. Condensation of mentioned resulted in diamide **133**, which after esterification with diazomethane, followed by reduction with LiAlH_4_ and aqueous workup, gave the desired quinazolinone scaffold **134**^104^. Oxidation to carboxylic acid **135** and amide bond formation with appropriately protected aspartic or glutamic acid (EDCI/HOBt technique) led to diester **136**, which was subsequently aminolysis to **137** with hydrazine. Eventually, conjugation with appropriate aromatic aldehyde resulted in final compounds **138–145** (Scheme 31)^103^. The obtained derivatives exhibited promising antibacterial and antifungal activities in disc diffusion tests. Molecular docking studies on the GlcN-6-P synthase matrix (pdbid: 2vf5) showed that the proposed compounds interacted with the ISOM domain active site, including Glu488, Ala602, Ser401, Gln348, Ser303, Gly301 and Thr352 residues. The docking scores obtained for **138–145** ranged between −8.969 and −12.238100.

**Scheme 32.**
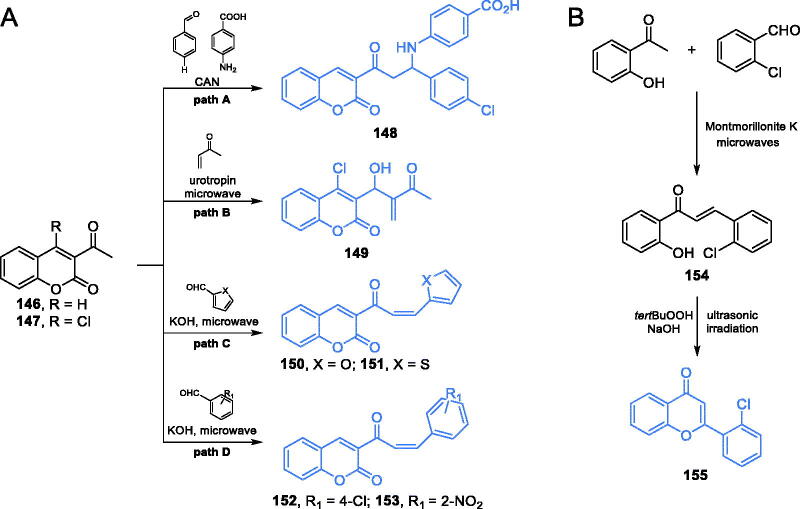
(A) Syntheses of coumarin based potential inhibitors of GlcN-6-P synthase, according to Kenchappa (path A)^92^, Kumar (path B)^107^ and Helmy *et al*. (path C)^108^. (B) Synthesis of 4-chromone-based inhibitor of GlcN-6-P synthase, according to Devi *et al*.^109^

Coumarin is a natural compound known for its broad spectrum of biological and pharmacological activities, such as antifungal, antioxidant, antibacterial^105^^,^^106^ or anticancer^106^ properties. Over the years, many pharmacologically potent derivatives based on the coumarin structure were proposed. Kenchappa *et al*.^92^ described the synthesis of some novel coumarin derivatives *via* three component Mannich reaction in the presence of CAN as a catalyst. The reaction occurred between 3-acetyl coumarin **146** (Scheme 32A, path A), 4-chlorobenzaldehyde and 4-aminobenzoic acid. Evaluation of antifungal properties of thus obtained compound **148** revealed its good inhibitory effect on *Aspergillus flavus* and *Chrisosporium keratinophilum* growth, with MIC of 40 µg/mL. Molecular docking to GlcN-6-P synthase (pdbid: 2vf5) confirmed, that **148** had an ability of binding to the active centre of ISOM domain of the target enzyme *via* hydrogen bonds with Ser347 and one hydrogen bond with Cys300 (estimated *K*_i_ = 22.19 µM)^92^.

Another potential GlcN-6-P synthase inhibitor based on the coumarin derivatives was proposed by Kumar *et al*.^107^ The authors described the synthesis of Baylis-Hillman adducts of coumarin, according to which a coumarin derivative **147** (Scheme 32A, path B) reacted with an appropriate alkene in the presence of 5 mol% of urotropine as a catalyst. Moreover, all compounds were prepared by the use of ionic liquids [BMIM]BF_4_ and [BIPIM]BF_4_ and under microwave irradiation. Ionic liquids, aside from being green chemical solvents, also played a catalytic role. The synthesised compound **149** was supposed to be a good GlcN-6-P synthase inhibitor, based on the results of molecular docking studies and antibacterial activity evaluation. This compound exhibited some growth inhibitory effect against Gram-positive bacteria *S. aureus* and *B. subtilis*. Molecular docking simulations of **149** interactions with GlcN-6-P synthase (pdbid: 2vf5) exhibited interactions with Cys300, Thr352, Gly301, Val605, Ser604, Lys603, and Ala602 residues at the active centre of the ISOM domain, with a binding energy of −4.747 kcal/mol^107^.

The microwave method in the synthesis of coumarin derivatives was also applied by Helmy *et al*.^108^ as a greener approach in organic synthesis, compared to the conventional method. The one-step synthesis involved a reaction between 3-acetyl coumarin **147** and an appropriate carbonyl compound. Due to the presence of the strong base, an aldol condensation occurred, resulting in products **150–151** and **152–153**^108^. *In vitro* growth, inhibitory activity against four selected strains (Gram-positive bacteria *S. aureus* and *B. subtilis* and Gram-negative bacteria *E. coli* and *Proteus vulgaris*) revealed that compound **153** (Scheme 32A, path D) emerged as the most active, especially against *E. coli* (MIC = 98 µg/mL, compared to amikacin, MIC = 121 µg/mL). Coumarin derivative **151** (Scheme 32A, path C) also showed good activity against all the tested microorganisms and was found to be the most active against *B. subtilis* (MIC of 123 µg/mL, compared to amikacin, MIC = 98 µg/mL). Both compounds showed the lowest docking scores (−13.9 for **151** and −17.72 for **153**) and interaction with Ala602 at the active site of the ISOM domain (pdbid: 2vf5)^108^.

Devi and co-workers^109^ reported a 4-chromone-based compound **155** (Scheme 32B) as an antimicrobial agent. According to the authors, the synthesis of **155** involving microwave and ultrasonic irradiation should be considered a “green” one. The chalcone **154**, obtained from 2-hydroxyacetophenone and 2-chlorobenzaldehyde in the presence of Montmorillonite K under microwave irradiation, was cyclised to 4-chromone **155** in an alkaline environment of NaOH and the presence of *tert*-butyl hydroperoxide under ultrasonic irradiation (Scheme 32B)^109^. In a disc diffusion test, derivative **155** exhibited antifungal activity, comparable to that of the standard drug, fluconazole. Molecular docking studies on GlcN-6-P synthase (podbid: 2vf5) showed that **155** interacted with the ISOM domain active site (docking score −89.68) via hydrogen bond formation with Glu384, Ser349 and Lys603 residues^109^.

**Scheme 33.**
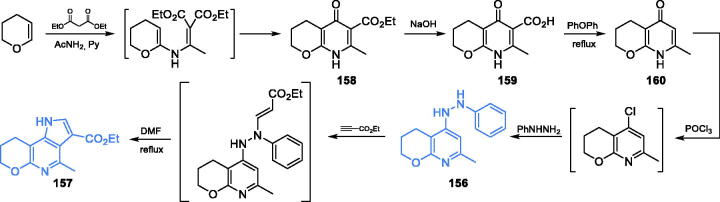
Synthesis of potential GlcN-6-P synthase inhibitors, according to Elkanzi *et al*.^110^

Promising antimicrobial activity was observed for bicyclic **156** and tricyclic **157** derivatives (Scheme 33) incorporating pyridine- and indole-based scaffolds. According to Elkanzi and co-workers^110^, the synthesis of **156** and **157** started with condensation of 3,4-dihydro-2*H*-pyran, diethyl malonate and acetamide, which resulted in the production of a nitrogen-containing ring of derivative **158**. Subsequent basic hydrolysis gave carboxylic acid **159**, which was decarboxylated to **160** by heating in diphenyl ether. Chlorination of **160** with POCl_3_ followed by a substitution reaction with phenylhydrazine gave inhibitor **156** that underwent the coupling reaction with ethyl propiolate followed by cyclisation to inhibitor **157** (Scheme 33)^110^. Compound **156** which was an intermediate in the synthesis of **157** exhibited comparable antifungal activity as that showed by the standard drug ketoconazole towards *Candida* sp. In the disc diffusion test, derivative **157** occurred to be an effective antibacterial agent, with the activity comparable with that of the standard ampicillin. Molecular docking experiments accomplished on the GAH domain of GlcN-6-P synthase matrix (pdbid: 1xff) revealed that both derivatives **156** and **157** could bind at the GAH active site by hydrogen bonding with Gly99 for **156** (binding energy −20.52 kcal/mol) and Thr76 and His97 in the case of derivative **157** (binding energy −19.4 kcal/mol)^110^.

### 6.5. Complexes of d and f block elements

**Scheme 34.**
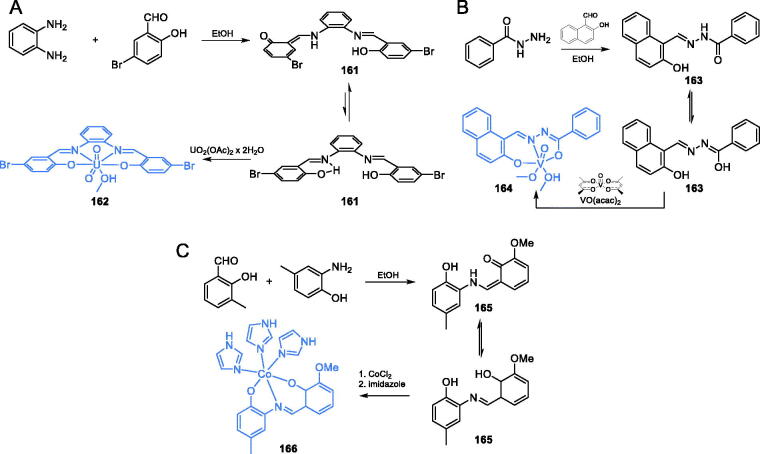
Syntheses of: (A) uranyl(VI) complex; (B) vanadium(V) complex; (C) cobalt(II) complex with antimicrobial activity, according to Ebrahimipour *et al*.^113–115^

Transition metal complexes of some organic compounds were reported as antifungal, antibacterial, analgesic or anti-inflammatory agents^111^^,^^112^. Ebrahimipour *et al*. characterised an antimicrobial activity of uranyl(VI) Schiff base complexes and suggested this activity as a consequence of GlcN-6-P synthase inhibition^113^. The synthesis of complex **162** started with the formation of ligand **161** which was obtained by condensation of 1,2-diaminobenzene with 5-bromo-2-hydroxybenzaldehyde. Ligand **161** was subjected to complexation with a methanolic solution of UO_2_(OAc)_2_×2H_2_O, giving target uranyl complex **162** (Scheme 34A)^113^. Complex **162** (Scheme 34A) exhibited some activity against Gram-negative bacteria, with MIC values of 0.937–3.75 mg/mL, and against *C. albicans* (MIC = 7.5 mg/mL). *In silico* study done on GlcN-6-P synthase matrix (pdbid: 2vfc)revealed that the uranyl(VI) complex **162** may be considered a good inhibitor of GlcN-6-P synthase, assuming its binding at the active site of ISOM domain, with a binding energy of −8.9 kcal/mol^113^.

The same research group also synthesised and evaluated the antimicrobial activity of three tridentate oxido-vanadium(V) complexes, including **164** (Scheme 34B). The synthesis of the ligand for complex **164** was a one-step reaction depending on the condensation of benzohydrazide and 2-hydroxynaphthaldehyde in ethanol, resulting in hydrazone **163**. The final addition of VO(acac)_2_ in methanol to **163** gave the final complex **164** (Scheme 34B)^114^. *In vitro* experiments showed that all complexes exhibited significant activity against both Gram-positive and Gram-negative bacteria, as well as against methicillin-resistant *S. aureus* (MIC = 19.5 µg/mL). Docking studies proceeded on GlcN-6-P synthase matrix (pdbid: 2vf5) suggesting that complex **164** should be a good inhibitor of this enzyme, binding at the ISOM domain. The inhibition constant and binding energy were estimated as 123.33 µM and −5.33 kcal/mol, respectively^114^.

In another work, Ebrahimipour *et al*.^115^ reported novel cobalt(III) complexes **166** incorporating imidazole and 2-((3-methoxy-2-oxidobenzylidene)amino)-4-methylphenolate ligand (Scheme 34C). Synthesis of **165** was accomplished by condensation of 2-hydroxy-3-methoxybenzaldehyde and 2-amino-4-methylphenol. Subsequent sequential complexation with CoCl_2_ and imidazole resulted in target complex **166** (Scheme 34C)^115^. The obtained complex **166** was tested against some Gram-negative and Gram-positive bacteria as well as against human pathogenic yeasts *C. albicans*. Complex **166** exhibited significant activity against all tested strains, with MIC values ranging between 0.78 and 3.125 mg/mL. Molecular docking studies to GlcN-6-P synthase (pdbid: 2vf5) revealed binding of **166** to the ISOM domain active site *via* interactions with Cys300, Gly301, Leu484, Leu601, Glu488, and Lys487 residues, with a binding energy of −6.69 kcal/mol^115^.

**Scheme 35.**
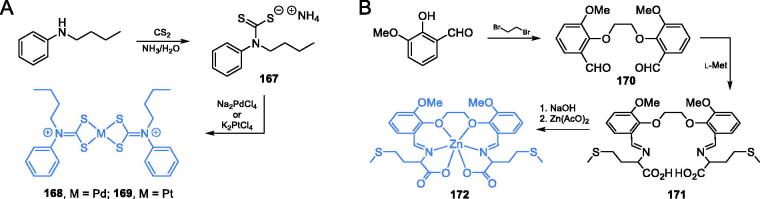
Synthesis of metal complexes, potential GlcN-6-P synthase inhibitors, according to (A) Onwudiwe *et al*.^116^ and (B) Wang *et al*.^118^

Palladium(II) and platinum(II) complexes (**168** and **169**) of *N*-butyl-*N*-phenyldithiocarbamate (Scheme 35A) were reported by Onwudiwe *et al*.^116^ as potential GlcN-6-P synthase inhibitors. The synthesis of target complexes required for *N*-butyl-*N*-phenyldithiocarbamate ligand **167**, which was obtained as ammonium salt from *N*-butylaniline and carbon disulphide in ammonia condition. Subsequent treatment of ligand **167** with Na_2_PdCl_4_ or K_2_PtCl_4_ in aqueous media led to desired palladium and platinum complexes **168** and **169** (Scheme 35A)^116^^,^^117^. The antimicrobial activity of the mentioned complexes was tested against two bacterial (*E. coli* and *S. aureus*) and two fungal (*A. flavus* and *Fasiparium exosporium*) strains. The results showed that both palladium and platinum complexes exhibited some antifungal activity (MIC = 50–100 µg/mL), comparable to that of ketoconazole (MIC = 65–80 µg/mL). *In silico* docking experiments on GlcN-6-P synthase (pdbid: 2vf5) revealed potential strong binding interactions with the ISOM domain active site by interaction with Thr302, Gln348, Ser303, Ala400, and Val605 residues. The binding energies for **168** and **169** were estimated as −6.113 and −6.54 kcal/mol, respectively^116^.

The research group of Wang proposed three zinc(II) complexes with a sexidentate bis-Schiff base ligand^118^. The ligand **171** was prepared from 2-hydroxy-3-methoxybenzaldehyde, 1,2-dibromoethane and l-methionine in two synthetic steps. Firstly, the benzaldehyde derivative was condensed with 1,2-dibrompethane resulting in dialdehyde **170** and then imine bond formation was accomplished with l-methionine leading to ligand **171**. The target complex **172** was obtained by the addition of zinc acetate to the alkaline solution of ligand **171** (Scheme 35B)^118^. Obtained complexes were tested against *E. coli* and *S. aureus* by the agar-well diffusion method. Complex **172** (Scheme 35B) exhibited good antimicrobial activity against both bacterial strains with the average diameter of the inhibition zone comparable to that of ampicillin. The docking simulation accomplished on the GlcN-6-P synthase matrix (pdbid: 2vf5) showed that complex **172** was well embedded into the active site of the ISOM domain and interacted *via* H-bonding with Thr302 and Val605 residues, with the binding energy estimated as −9.97 kcal/mol^118^.

## 7. Inhibitors binding outside the active centres of GlcN-6-P synthase

One of the two products of the reaction catalysed by GlcN-6-P synthase, namely d-glucosamine-6-P, is a natural inhibitor of the bacterial version of this enzyme. On the other hand, eukaryotic GlcN-6-P synthase is a subject of feedback inhibition by UDP-GlcNAc^18^. The UDP-GlcNAc binding site is localised at ISOM domain but outside the active centre^119^. Other binding sites for potential inhibitors are the intramolecular channel and the contact areas between two dimers constituting a tetrameric structure of the eukaryotic GlcN-6-P synthase.

**Scheme 36.**
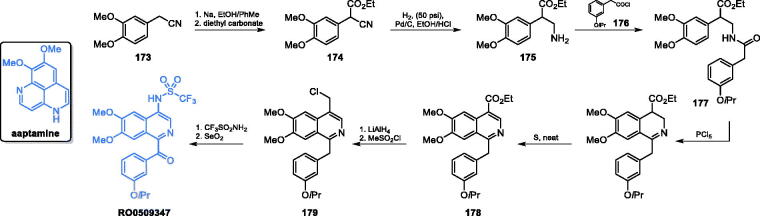
Structure of aaptamine and synthesis of **RO0509347** developed at Hoffman La-Roche Inc.^122^

Aaptamine (Scheme 36), a heterocyclic compound isolated from the sea sponge *Aaptos aaptos*, was found to be an inhibitor of GlcN-6-P synthase, with IC_S0_ = 120 µM^120^. Several aaptamine derivatives exhibited antifungal activity^121^. A much stronger inhibitor of human GlcN-6-P synthase, compound **RO0509347** (IC_50_ = 1 µM) resulted from the high throughput screening and subsequent hit identification and optimisation at Hoffman La-Roche Inc. That compound demonstrated significant efficacy in reducing glucose excursion in oral glucose tolerance tests in diet-induced obesity mice^122^. Synthesis of that compound started with commercially available (3,4-dimethoxy)acetonitrile **173**, which underwent base-catalysed condensation with diethyl carbonate, resulting in α-cyanoester **174**. Subsequent reduction of nitrile moiety with gaseous hydrogen on Pd/C catalyst under elevated pressure gave amine **175**, which after acylation with acyl chloride **176**, resulted in amide **177**. The formation of the isoquinoline ring of **178** was accomplished in the Bischler-Napieralski manner by treatment of **177** with PCl_5_, followed by aromatisation reaction utilising neat sulphur. Then, the obtained isoquinoline ester **178** was converted to alkyl halide **179** in a two-step manner, depending on the reduction of ester moiety with LiAlH_4_, followed by the treatment with methanesulfonyl chloride. Subsequent substitution of chloride atom with trifluoromethanesulfonyl amide and oxidation with SeO_2_ gave the final compound **RO0509347** (Scheme 36)^122^.

**Figure 2.**
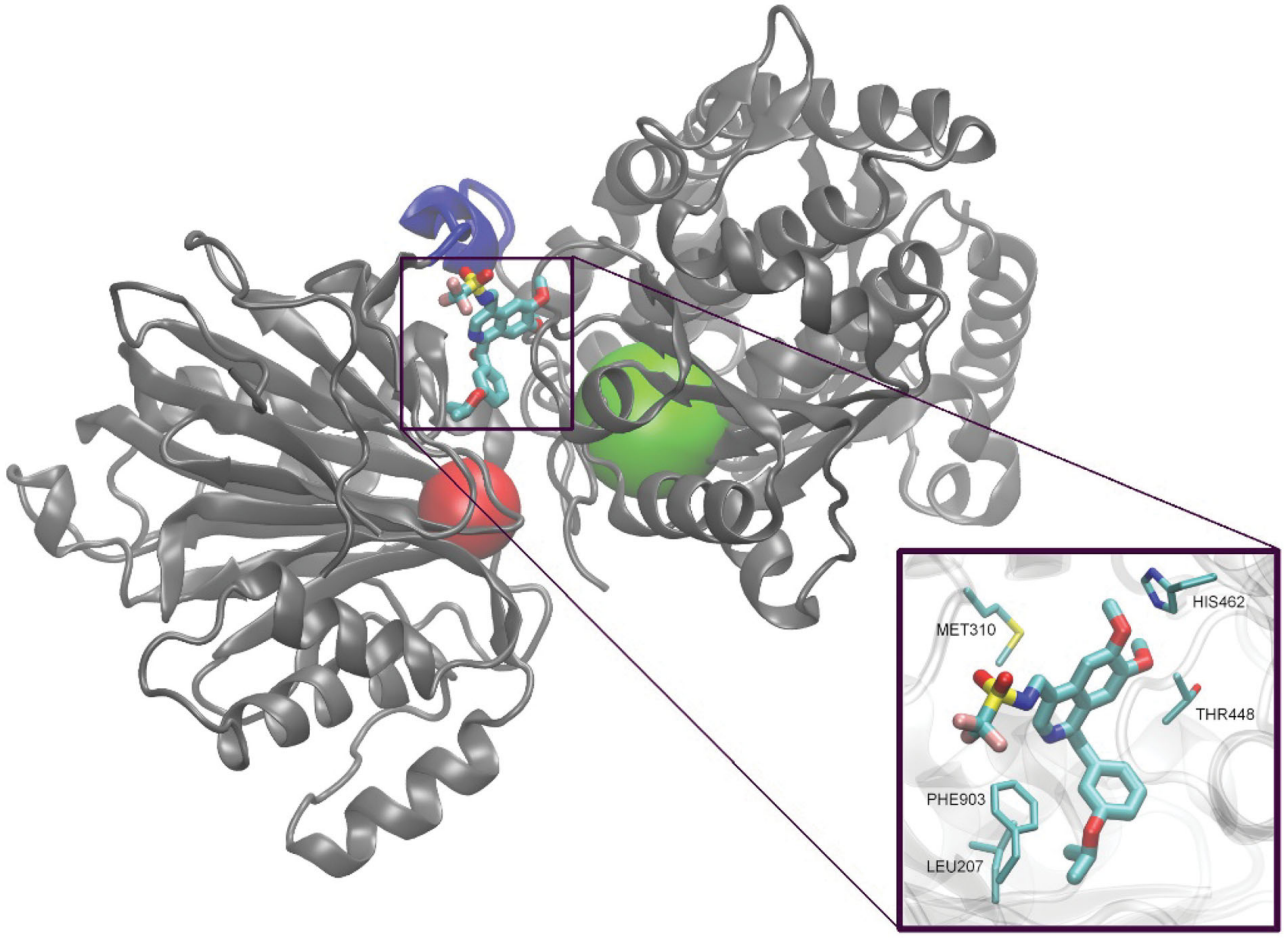
The hypothetical complex of **RO0509347** with human GlcN-6-P synthase. Drawing based on the results of docking calculations to the hGFAT2 matrix (pdbid: 6r4f)^123^, performed with the use of Autodock 4.2, according to the procedure described previously^124^. A single subunit of the tetrameric enzyme is shown, with GAH and ISOM active centres indicated as red and green spheres respectively. A flexible linker joining both domains of the enzyme is coloured blue.

The binding site for **RO0509347** at GlcN-6-P synthase has not been unequivocally identified. However, a molecular docking of this compound to the human GlcN-6-P synthase (hGFAT2) matrix, results of which are shown in Figure 2, revealed the cavity located in the vicinity of the intramolecular channel connecting GAH and ISOM domains as the most probable binding site. A putative binding site is located in a narrow cleft between the two domains near the interdomain linker. Since the precise interdomain communication is crucial for the catalysis, despite the fact that the bound inhibitor does not directly interfere with any of the enzyme active sites, binding the ligand at this site should hinder the interdomain communication and thus disturb catalytic reaction. In the bound ligand conformation, the polar sulphonamide group sticks out of the cleft to the aqueous surrounding, the isoquinoline moiety participates in the favourable MET-π interactions, while the aliphatic part of isopropoxy moiety is trapped in a small hydrophobic pocket formed by Phe903 and Leu207. This pose and interactions of the bound ligand correlate well with the SAR data for this group of compounds^122^.

### 7.1. Inhibitors based on five-membered ring scaffolds

**Scheme 37.**
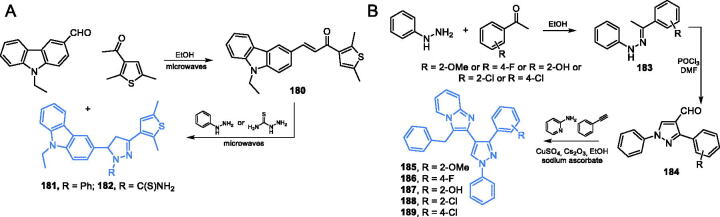
(A) Synthesis of 1,2-diazole-based inhibitors of GlcN-6-P synthase according to Khan *et al*.^125^ (B) Synthesis of trisubstituted 1,2-diazole-based inhibitors of GlcN-6-P synthase according to Ebenezer *et al*.^126^

Khan and co-workers^125^ described trisubstituted pyrazole-based potential inhibitors of GlcN-6-P synthase. The microwave-assisted synthesis was applied to the production of final inhibitors, as well as intermediate chalcone **180**, which was obtained by aldol reaction between 3-acetyl-2,5-dimethylthiphene and 9-ethyl-9*H*-carbazole-3-carbaldehyde. The eventual formation of a 1,2-diazole-based ring by cyclisation reaction with appropriate hydrazine derivative resulted in final inhibitors **181** and **182** (Scheme 37A)^125^. Derivatives **181** and **182** showed antibacterial (against *S. aureus, S. pyogenes, S. typhimurium* and *E. coli*) activity, with MIC values identical or twice the time lower that of the standard drug chloramphenicol (32 µg/mL). Molecular docking experiments accomplished on *E. coli* GlcN-6-P synthase (pdbid: 2vf4) revealed that the obtained compounds may bind to the enzyme ISOM domain outside the active site, *via* interaction with Asp474, Ser310, Trp312, Ala404 and Glu569 residues. Calculated binding energies for **181** and **182** were −8.5 and −9.2 kcal/mol, respectively^125^.

A set of 1,2-diazol-based compounds were synthesised and biologically evaluated by Ebenezer and co-workers^126^. The synthesis of inhibitors began with an initial condensation of phenylhydrazine and appropriately substituted acetophenone giving phenyl hydrazone **183**, which subsequently underwent the Vilsmeier-Haack formylation followed by cyclisation, using a mixture of POCl_3_ and dimethylformamide. The obtained 1,2-diazole **184** was allowed to undergo a one-pot three-component reaction with 2-aminopyridine and phenylacetylene, catalysed by CuSO_4_/sodium ascorbate to produce the final putativeinhibitors **185–189** (Scheme 37B)^126^. Those five derivatives exhibited good bactericidal activity against both Gram-positive and Gram-negative bacteria. Moreover, derivative **186** showed significant activity against MRSA, reaching the minimum bactericidal concentration (MBC) value of 2.5 µg/mL, compared to 1.84 µg/mL of the standard drug ciprofloxacin. The *in silico* investigations proceeded on GlcN-6-P synthase (pdbid: 1jxa) suggesting that antibacterial activity of **185–189** could be due to their binding outside the active sites of GlcN-6-P synthase, mainly by interaction with Arg21, Arg22, Glu24, Tyr251, and Ile397 residues. The binding energies for proposed compounds ranged between −9.5 and −10.5 kcal/mol^126^.

**Scheme 38.**
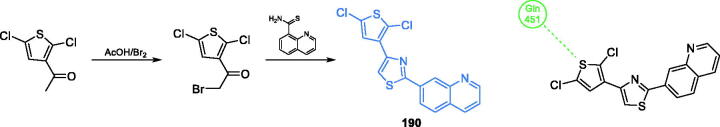
(A) Syntheses of a possible GlcN-6-P inhibitor, according to Sarojini *et al*.^127^^,^^128^ and its predicted binding mode to GlcN-6-P synthase; H-bonds are shown by dashed lines.

Sarojini et al. described _the_ synthesis of new series of 2-substituted-4–(2,5-dichloro thienyl)-1,3-thiazoles^127^ (Scheme 38) based on thiazole derivatives proposed by Narayana et al.^128^, some of which showed excellent antifungal and antibacterial activity. The 2,5-dichloro substituted thienyl derivative was synthesised by the reaction of 2-bromo-1–(2,5-dichlorothien-3-yl) ethenone with 8-quinolyl substituted thioamide. Nucleophilic attack of the amino group of thioamide on ethenone derivative was followed by a second ring formation. An intermediate product was obtained through bromination of 1–(2,5-dichlorothien-3-yl) ethanone in acetic acid (Scheme 38)^127^^,^^128^. While most of the newly synthesised thienyl derivatives did not exhibit satisfactory antimicrobial activity, one of them **−190** emerged as highly active against all tested microorganisms, with MIC values ranging between 6.25 and 12.5 µg/ml (6.25 µg/ml for ampicillin). Molecular docking to GlcN-6-P synthase matrix (pdbid: 1jxa) revealed that **190** may be a good inhibitor of this enzyme, as it is expected to bind to Gln451 residue outside the active site (estimated *K*_i_ = 0.957 µM)^127^.

**Scheme 39.**
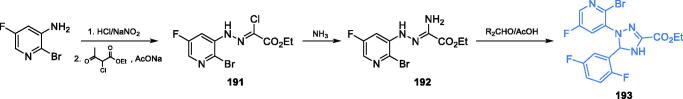
Synthesis of trisubstituted 1,2,4-triazole as a potential inhibitor of GlcN-6-P synthase, according to Krishna *et al*.^129^

Another triazole derivative was designed, synthesised and described by Krishna *et al*.^129^ The proposed compound was synthesised in four steps manner, beginning with the conversion of 3-amino-2-bromo-5-chloropyridine to imine **191** by treatment with an aqueous solution of hydrochloric acid and sodium nitrite, followed by addition of ethyl 2-chloro acetoacetate and sodium acetate. Subsequent treatment of **191** with gaseous ammonia resulted in azaenol product **192**, which was cyclized to final inhibitor **193** by condensation with 2,5-difluorobenzaldehyde (Scheme 39)^129^. Compound **193** was subjected to *in vitro, in vivo* and *in silico* biological activity screening, including antibacterial, antiproliferative and anti-inflammatory activity determination. Derivative **193** emerged as an agent effective against all tested bacterial strains in disc diffusion tests, compared to the standard drug nitrofurazone. Molecular docking to GlcN-6-P synthase (pdbid: 1jxa) revealed that **193** could bind to the enzyme outside the active centres, with minimum docking energy of −175.9 kJ/mol^129^.

**Scheme 40.**
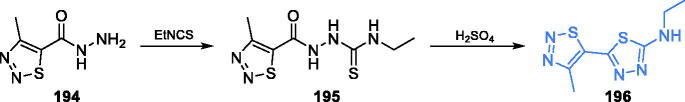
Synthesis of the 1,3,4-thiadiazole derivative as a putative inhibitor of GlcN-6-P synthase, according to Siwek *et al*.^130^

Sarojini results were mentioned by Siwek et al.^130^ in their work on 1,3,4-thiadiazole and s-triazole derivatives as potent GlcN-6-P synthase inhibitors. The synthesis of proposed structures was published in a previous work of the same group on s*-*triazoles as antibacterial agents^131^. The thiadiazole-based derivative was prepared using as a starting material 4-methyl-1,2,3-thiadiazole-5-carboxylic acid hydrazide **194** (Scheme 40). The reaction of the **194** with ethyl isothiocyanate gave thiosemicarbazide **195**, which subsequently was treated with sulphuric acid to obtain compound **196**. The proposed compound, exhibited some antimicrobial inhibitory effect, exclusively against *Candida* spp. Comparing the results of the biological activity with molecular docking studies, **196** may be considered a potential inhibitor of GlcN-6-P synthase. This derivative showed minimal binding energy of −1.7 kcal/mol in molecular docking at the GAH domain (pdbid: 1xff). Despite the fact that most of the prepared derivatives did not show antimicrobial effect, the presented results might be useful, for example as a reference set of inactive structures in the construction of QSAR models^130^.

**Scheme 41.**
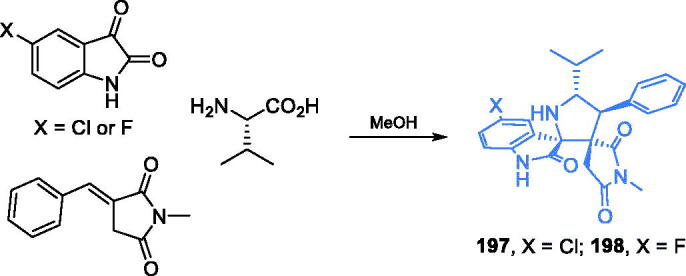
Synthesis of spiro pyrrolidine-based putative inhibitor of GlcN-6-P synthase, according to Askri *et al*.^132^

Recently Askri and co-workers^132^ reported antimicrobial activity of some spiro-derivatives based on pyrrolidine scaffold. A three-component cascade reaction of (*E*)-3-arylidene-1-methylpyrrolidine-2–5-diones, l-valine and isatine derivatives, involving 1,3-dipolar cycloaddition was applied for the synthesis of **197** and **198** (Scheme 41)^132^. Obtained compounds exhibited good activity (MIC = 3.9 µg/mL) against *S. aureus,* compared to that of the standard drug, tetracycline (24 µg/mL) and moderate activity against *C. albicans* (MIC = 78 µg/mL). Molecular docking experiments accomplished on GlcN-6-P synthase (pdbid: 1jxa) revealed that derivatives **197** and **198** interacted with several amino acid residues *via* H-bonding, including Asp192 and Glu351 in the case of **197** (docking score −15.33) and Pro377 in the case of **198** (docking score −13.4)^132^.

### 7.2. Inhibitors based on six-membered ring scaffolds

**Scheme 42.**
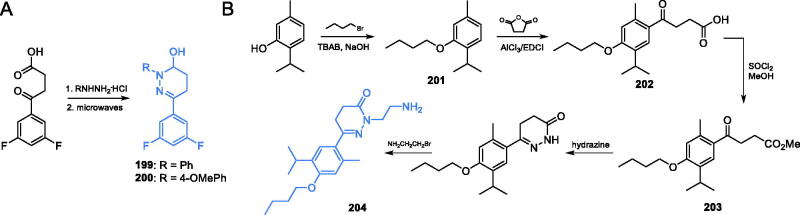
(A) Synthesis of fluorinated pyridazinone derivatives as a potential inhibitor of GlcN-6-P synthase, according to Sowmya *et al*.^133^ (B) Synthesis of pyridazinone derivatives as a putative inhibitor of GlcN-6-P synthase, according to Nagle *et al*.^134^

Sowmya et al. proposed a synthesis of novel fluorinated pyridazinone derivatives^133^. The synthesis involved microwave irradiation, which made possible application of the solvent free conditions. Synthesis of six 3-(2*H*)-pyridazinone derivatives started with grounding up 4–(3,5-difluorophenyl)-butanoic acid and an appropriate hydrazine hydrochloride derivative in the presence of a catalytic amount of acetic acid. The resulting solid was subsequently exposed onto a microwave initiator. The use of microwaves resulted in high yields, over 85% for all prepared compounds and the duration of each synthesis did not exceed 10 min. Moreover, solvent-free conditions made that synthesis more environmentally friendly^133^. Compounds **199** and **200** (Scheme 42A) were the most active against three bacterial strains (*E. coli, B. cereus, S. aureus*) compared to streptomycin, with diameters of the zones of inhibition obtained for **199** and **200** ranging between 65–76% and 54–65%, respectively, of that of the standard drug. The molecular docking studies confirmed that both compounds are potential inhibitors of GlcN-6-P synthase (PDB 1XFF). Compound **199** showed three interactions, with Pro198, Thr200 and Arg202, while **200** demonstrated interactions with Arg201 and Thr200 at GAH, outside the active centre of this domain^133^.

Another synthesis of compounds based on the pyridazinone ring was described by Nagle *et al*.^134^ The proposed diazine derivatives contained a thymol group in their structure, which may contribute to the overall biological activity of the final compounds since thymol is known for a wide spectrum of biological properties^135^^,^^136^. The first step of the synthesis involved a nucleophilic attack of the thymol hydroxyl group on *n*-butyl bromide in the presence of a strong base and phase transition catalyst, TBAB^137^. The resulting ether **201** was subsequently treated with succinic anhydride and aluminium chloride with EDCI. The acylation of the aromatic ring was followed by esterification, which occurred for the newly introduced carboxyl moiety of **202**, resulting in ester **203**. Subsequently, pyridazinone ring was formed by the use of hydrazine hydrate and then the alkyl group was introduced on nitrogen atom in the presence of sodium hydride, affording the final compound **204** (Scheme 42B)^134^. The obtained derivative exhibited relatively good antimicrobial activity. Molecular docking to GlcN-6-P (pdbid: 1jxa) revealed that **204** may bind at the ISOM domain, interacting with Glu534 and Glu79, outside the active centre^134^.

### 7.3. Inhibitors based on naphthalene-based scaffolds

**Scheme 43.**
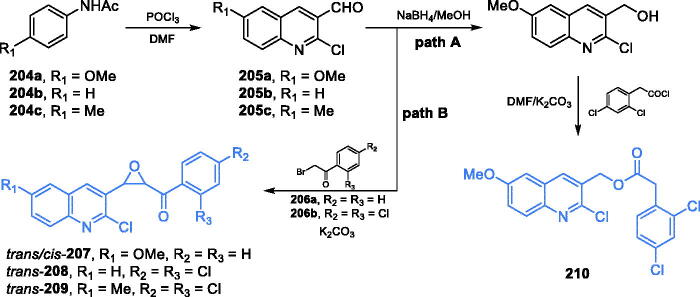
Synthesis of 2-chloroquinolin-3-yl ester derivatives as potential GlcN-6-P inhibitors, according to Tabassum *et al*. (path A)^139^. Synthesis of quinoline-based epoxides, according to Preveena *et al*. (path B)^138^.

Preveena and co-workers^138^ reported a series of naphthalene-based compounds with highly promising pharmacological properties. The proposed synthesis started from appropriate *para*-substituted acetanilide **204a-c**, which underwent a cyclisation reaction in DMF/POCl_3_ conditions. The obtained quinoline derivatives **205a-c** were subjected to Darzens condensation with 2,4-disubstituted-α-bromoketone **206a-b** under mild basic conditions, resulting in final epoxides **207–209**. In the case of **208** and **209**, predominantly *trans* isomers were obtained, with small quantities of the corresponding *cis* isomers. Derivative **207** was obtained as an almost equimolar mixture of *trans* and *cis* isomers (Scheme 43, path B)^138^. Obtained compounds exhibited good antibacterial activity against *B. subtilis* (**207**), *E. coli* (**208**), and *S. aureus* (**209**), comparable with that of the standard antibiotics (ofloxacin and ampicillin). Moreover, derivatives **208** and **209** showed the best antifungal activity, similar to that of the standard drug, fluconazole. Both *cis* and *trans* isomers of **207–209** exhibited similar antimicrobial activity. The docking experiments indicated good interaction of mentioned agents with the GlcN-6-P synthase GAH domain (pdbid: 1xff) outside the active site. The *trans* isomers created more hydrogen bonds with the GAH domain in comparison to the *cis* ones. In the case of *trans* isomers, H-bonding was observed with Arg201, Asp11, Gly66, Thr200 and Arg22 residues, while the *cis* ones created H-bonds with Glu14, Arg217, Gly66 and Asp11 residues. The binding energies for **207–209** ranged between −7.96 and −8.35 kcal/mol^138^.

Another series of quinoline derivatives were synthesised and described by Tabassum *et al*.^139^ The novel 2-chloroquinolin-3-yl ester derivative **210** was obtained in a manner similar to that of **205a–c**, using Veismeier-Haack cyclisation of appropriate acetanilide derivative to quinolone derivatives. Subsequently, the resulting aldehyde **205a** was reduced with sodium borohydride and the formed alcohol was eventually esterified by chlorinated phenylacetic acid chloride under alkaline conditions (Scheme 43, path A)^139^. Among all prepared quinoline derivatives, the best antimicrobial activity in disc diffusion tests was exhibited by compound **210** (Scheme 43, path A). Docking to GlcN-6-P synthase matrix (pdbid: 1xff) revealed binding of **210** due to its interaction with Met184, Arg10, Arg216 and Arg217 residues outside the active site of the GAH domain (estimated *K*_i_ = 0.0764 µM)^139^.

**Scheme 44.**
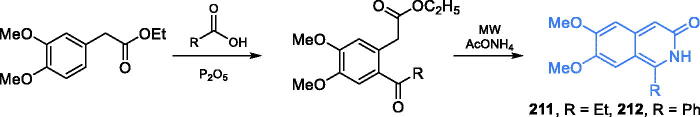
Synthesis of isoquinoline derivatives as potential inhibitors of GlcN-6-P synthase, according to Borse *et al*.^140^

Borse *et al*.^140^ described the two-step synthesis of 12 isoquinoline derivatives. The first step of the synthesis involved a reaction between an appropriate carboxylic acid and ethyl 3,4-dimethoxyphenyl acetate, in the presence of phosphorous oxide. As a part of the second step, resulting intermediates were treated with ammonium acetate under solvent-free conditions, to be finally irradiated with microwaves (Scheme 44)^140^. All obtained products were found to exhibit moderate to good antimicrobial activity and compared to the results of the molecular docking study, suggesting that all derivatives may be considered good GlcN-6-P synthase inhibitors. However, compounds **211** and **212** emerged as the most promising antimicrobials among all tested isoquinoline derivatives. Compound **211** showed promising activity against *S. aureus* and *C. albicans* and **212** against *S. aureus* only. Molecular docking to the GlcN-6-P synthase matrix (pdbid: 1jxa) confirmed, that both compounds may be considered as potential GlcN-6-P synthase inhibitors, possibly binding to the ISOM domain (E = −109,41 kJ/mol for **211** and E = −135.48 kJ/mol for **212**)^140^.

## 8. Conclusions and perspectives

GlcN-6-P synthase is one of the enzymes most extensively studied as a molecular target for potential novel antimicrobial or antidiabetic drugs. Inhibitors targeting GAH or ISOM active sites, rationally designed or of natural origins, such as FMDP, DSOK, APO or ADGP are highly selective for GlcN-6-P synthase. However, most of them are hydrophilic compounds, poorly penetrating biological membranes. In consequence, their antimicrobial activity is low. Hopefully, their antimicrobial potential could be improved upon conversion into derivatives of the pro-drug type, especially by employing molecular nanocarriers^141–143^ that could ensure efficient delivery of nanocarrier: GlcN-6-P synthase inhibitor to the microbial cell interior. Nevertheless, cleavable conjugates, able to release the active inhibitor in the cytosol may have potential as antimicrobial drug candidates of a broad spectrum, covering human pathogenic bacteria and fungi.

A huge number of heterocyclic compounds exhibiting antimicrobial activity have been reported as possible GlcN-6-P inhibitors, based on the results of their molecular docking into bacterial GlcN-6-P synthase matrix. In some cases, especially for compounds **55**, **67c**, **74** and **113**, the calculated values of docking score, binding energy or inhibitory constants have suggested their strong enzyme inhibitory potential but only for **67c,** this potential has been confirmed by experimental data. Moreover, little if not at all is known about the selectivity of these compounds as GlcN-6-P synthase inhibitors and selective toxicity in the pathogenic microorganism: the human host system. Compounds, for which selective toxicity due to the GlcN-6-P synthase inhibition will be confirmed, are surely worth further investigating.

Very few confirmed GlcN-6-P synthase inhibitors bind outside the GAH and ISOM active sites. Among them, the aaptamine derivatives, such as **RO0509347**, presumably interfering with interdomain communication between GAH and ISOM, seem especially interesting. Although originally developed as antidiabetics, they may also have potential as antimicrobials. This possibility should be thoroughly further examined.
